# Introduction of a SiFA Moiety into the D-Glutamate Chain of DOTA-PP-F11N Results in Radiohybrid-Based CCK-2R-Targeted Compounds with Improved Pharmacokinetics In Vivo

**DOI:** 10.3390/ph15121467

**Published:** 2022-11-25

**Authors:** Nadine Holzleitner, Thomas Günther, Roswitha Beck, Constantin Lapa, Hans-Jürgen Wester

**Affiliations:** 1 Pharmaceutical Radiochemistry, Technical University of Munich, 85748 Garching, Germany; 2 Nuclear Medicine, Faculty of Medicine, University of Augsburg, 86156 Augsburg, Germany

**Keywords:** cholecystokinin-2 receptor (CCK-2R), cholecystokinin-B receptor (CCK-BR), medullary thyroid cancer (MTC), minigastrin analogues, radiohybrid, rhCCK

## Abstract

In order to enable ^18^F- and ^177^Lu-labelling within the same molecule, we introduced a silicon-based fluoride acceptor (SiFA) into the hexa-D-glutamate chain of DOTA-PP-F11N. In addition, minigastrin analogues with a prolonged as well as *γ*-linked D-glutamate chain were synthesised and evaluated. CCK-2R affinity (*IC*_50_, AR42J cells) and lipophilicity (log*D*_7.4_) were determined. Biodistribution studies at 24 h post-injection (p.i.) and *µ*SPECT/CT imaging at 1, 4 and 24 h p.i. were carried out in AR42J tumour-bearing CB17-SCID mice. CCK-2R affinity of (*R*)-DOTAGA-rhCCK-1 to 18 was enhanced with increasing distance between the SiFA building block and the binding motif. Lipophilicity of [^177^Lu]Lu-(*R*)-DOTAGA-rhCCK-1 to 18 was higher compared to that of [^177^Lu]Lu-DOTA-PP-F11N and [^177^Lu]Lu-CP04. The respective *α*- and *γ*-linked rhCCK derivatives revealing the highest CCK-2R affinity were further evaluated in vivo. In comparison with [^177^Lu]Lu-DOTA-PP-F11N, [^177^Lu-]Lu-(*R*)-DOTAGA-rhCCK-9 and -16 exhibited three- to eight-fold increased activity levels in the tumour at 24 h p.i. However, activity levels in the kidneys were elevated as well. We could show that the introduction of a lipophilic SiFA moiety into the hydrophilic backbone of [^177^Lu]Lu-DOTA-PP-F11N led to a decelerated blood clearance and thus improved tumour retention. However, elevated kidney retention has to be addressed in future studies.

## 1. Introduction

Medullary thyroid carcinoma (MTC) constitutes for only 2–3% of all thyroid cancer cases and is therefore rather rare, but treatment options are limited: neither external beam radiation, nor conventional chemotherapy, nor radioiodine therapy are recommended, as all three concepts have not shown curative effects [1,2,3,4]. Tyrosine kinase inhibitors such as selpercatinib, vandetanib or cabozantinib are usually applied for systematic treatment but these agents are associated with distinct side effects such as renal toxicity, myelosuppression, arterial thromboembolism, hepatotoxicity, and muscle wasting [3,5].

Since Reubi et al. discovered that approximately 92% of all MTCs overexpress the cholecystokinin-2 receptor (CCK-2R), designing small compounds that address this target became attractive in combination with peptide receptor radionuclide imaging (PRRI) and therapy (PRRT) [6]. While first compounds were based on the structure of cholecystokinin, nowadays minigastrin-based ligands are clearly favoured because of their increased hydrophilicity [7]. However, early radiolabelled minigastrin analogues suffered from elevated activity levels in the kidneys, which hampered a potential therapeutic use [8,9].

An important step for the applicability of these minigastrin derivatives was the modification within the linker section, namely the substitution of the hexa-L-glutamate by a hexa-D-glutamate chain, which resulted in compounds such as CP04 and DOTA-PP-F11N, amongst others. The latter consists of a stabilised binding motif of seven amino acid with high CCK-2R affinity (*H*-Ala-Tyr-Gly-Trp-Nle-Asp-Phe-NH_2_), a hexa-D-glutamate linker and DOTA (1,4,7,10-tetraazacyclododecane-1,4,7,10- tetraacetic acid) as a chelator [10,11]. Nevertheless, due to the high hydrophilicity of [^177^Lu]Lu-DOTA-PP-F11N, first patient studies revealed a very rapid renal clearance already at 1 h post-injection (p.i.), which resulted in a median (interquartile range) absorbed tumour dose of only 0.88 Gy/GBq [12]. Moreover, none of the currently available CCK-2R-targeted compounds for clinical application bears an option for ^18^F-labelling.

Recently, radiohybrid (rh)-based prostate-specific membrane antigen (PSMA)-targeted compounds were developed by our group, implementing a new class of theranostic compounds. These compounds comprise a silicon-based fluoride acceptor (SiFA) moiety for rapid and facile ^18^F-fluorination via a ^18^F/^19^F isotopic exchange reaction and additionally contains a chelator for radiometallation (with ^68^Ga or ^177^Lu, amongst others). This concept results in a chemically identical pair of compounds (either ^18^F/non-radioactive metal or ^19^F/radiometal), which thus exhibits identical pharmacokinetics and can be used for either diagnostic or therapeutic applications [13,14].

**Figure 1:**
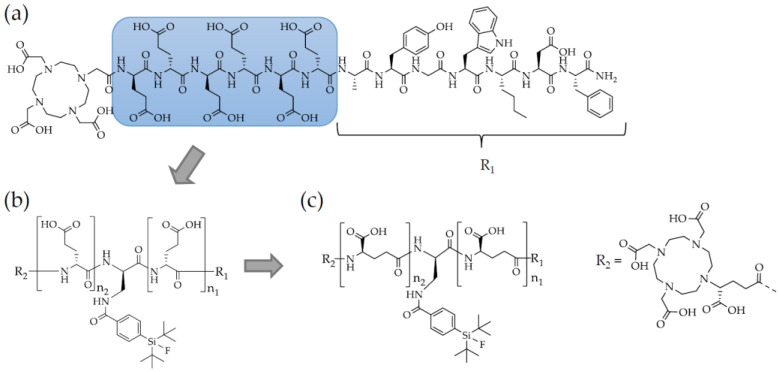
Structure of (**a**) DOTA-PP-F11N. (**b**) Structure of the rhCCK derivatives ((*R*)-DOTAGA-rhCCK-1-9) comprising a modified linker section generated via the introduction of a dap(SiFA) moiety into the D-glutamate chain ((*R*)-DOTAGA-rhCCK-1-7: n_1_ = 0 to 6 and n_2_ = 6 − n_1_; (*R*)-DOTAGA-rhCCK-8: n_1_ = 7 and n_2_ = 0; (*R*)-DOTAGA-rhCCK-9: n_1_ = 8 and n_2_ = 0) of DOTA-PP-F11N. (**c**) Structure of the rhCCK derivatives ((*R*)-DOTAGA-rhCCK-10-18) generated analogous to B but containing a *γ*-instead of an *α*-linked D-glutamate chain ((*R*)-DOTAGA-rhCCK-10-16: n_1_ = 0 to 6 and n_2_ = 6 − n_1_; (*R*)-DOTAGA-rhCCK-17: n_1_ = 7 and n_2_ = 0; (*R*)-DOTAGA-rhCCK-18: n_1_ = 8 and n_2_ = 0).

Given the promising clinical data of the rhPSMA derivatives [15,16,17,18], the aim of this study was to transfer the concept of rh-based compounds to minigastrin analogues. For this reason, we introduced a SiFA group into the highly hydrophilic hexa-D-glutamate chain of DOTA-PP-F11N via conjugation through a D-2,3-diaminopropionic acid (dap) moiety to generate a possibility for ^18^F-labelling and compensate for the high lipophilicity of the SiFA group. Moreover, DOTA was replaced by the more hydrophilic (*R*)-DOTAGA (2-(4,7,10-tris(carboxymethyl)-1,4,7,10-tetraazacyclododecan-1-6yl)pentanedioic acid) in all of our rhCCK derivatives. Besides the usually present *α*-linked poly-D-glutamate chain the rhCCK ligands were designed with a *γ*-linked poly-D-glutamate chain (Figure 1) as well and evaluated in state-of-the-art experiments.

## 2. Results

### 2.1. Synthesis and Radiolabelling

The uncomplexed ligands were synthesised via standard Fmoc-based SPPS, yielding 5–20% RP-HPLC purified precursors (chemical purity > 95%, determined by RP-HPLC at λ = 220 nm). Non-radioactive labelling proceeded quantitatively using a 2.5-fold excess of [^nat^Lu]LuCl_3_. No purification prior to affinity studies was performed, as the remaining free Lu^3+^ was shown to not affect affinity data [19]. ^177^Lu-labelling of all compounds was carried out manually resulting in quantitative radiochemical yields and purities of >95% as well as molar activities of 30 ± 10 GBq/µmol. After radiolabelling all peptides were used without further purification. Confirmation of peptide integrity and quality controls are depicted in the Supplementary Materials (Figures S1–S3).

### 2.2. In Vitro Characterisation

**Figure 2:**
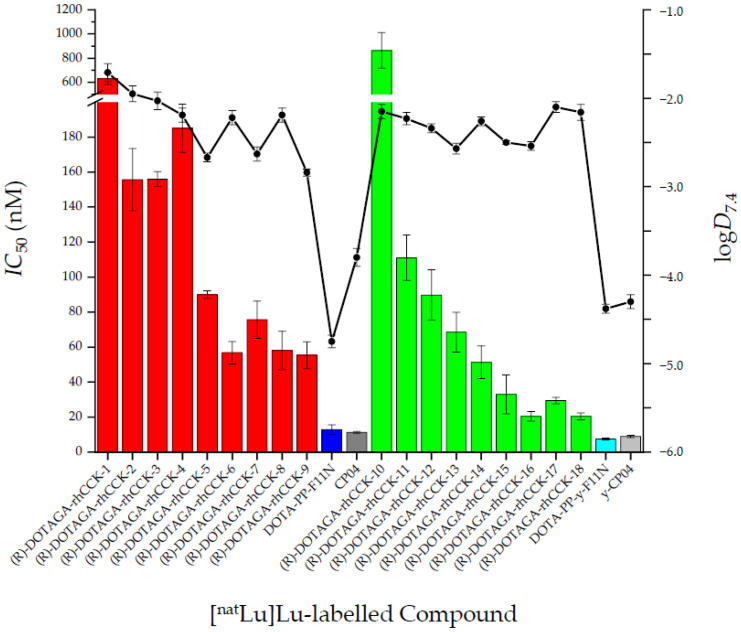
Affinity (*IC*_50_) and lipophilicity (log*D*_7.4_) data of the ^nat^Lu-labelled references DOTA-PP-F11N (dark blue) and CP04 (dark grey) compared to their *γ*-linked analogues DOTA-PP-*γ*-F11N (light blue) and *γ*-CP04 (light grey), the ^nat^Lu-labelled rhCCK derivatives comprising an *α*-linked D-glutamate chain ([^nat^Lu]Lu-(*R*)-DOTAGA-rhCCK-1-9, red) and the ^nat^Lu-labelled rhCCK derivatives containing a *γ*-linked D-glutamate chain ([^nat^Lu]Lu-(*R*)-DOTAGA-rhCCK-10-18, green). *IC*_50_ values were determined using AR42J cells (2.0 × 10^5^ cells per well) and [^177^Lu]Lu-DOTA-PP-F11N (0.3 pmol/well) as radiolabelled reference (3 h, 37 °C, RPMI 1640, 5 mm L-Gln, 5 mL non-essential amino acids (100×), 10% fetal calf serum (FCS) + 5% bovine serum albumin (BSA) (*v*/*v*)).

The affinity and lipophilicity data of all compounds are summarised in Figure 2 and Table S1.

In general, all ligands containing a *γ*-linked D-glutamate chain revealed a higher affinity towards CCK-2R compared to their *α*-linked counterparts, except for [^nat^Lu]Lu-**10**. Furthermore, a trend could be observed that with increasing distance of the dap(SiFA) moiety to the binding motif *IC*_50_ values decreased, irrespective whether the compounds are *α*- or *γ*-linked. Overall, [^nat^Lu]Lu-(*R*)-DOTAGA-rhCCK-16 and [^nat^Lu]Lu-(*R*)-DOTAGA-rhCCK-18 displayed the highest CCK-2R affinity among all SiFA-containing compounds. Nevertheless, all four reference ligands showed lower *IC*_50_ values, suggesting a negative impact of the SiFA unit irrespective of its position within the molecule.

All four reference ligands revealed a high hydrophilicity, exhibiting distribution coefficients (log*D*_7.4_) in a range of −4.8 and −3.8. Not surprisingly, the rhCCK derivatives comprising the lipophilic SiFA moiety displayed a distinctly higher lipophilicity (log*D*_7.4_ = −2.9 to −1.7).

**Figure 3:**
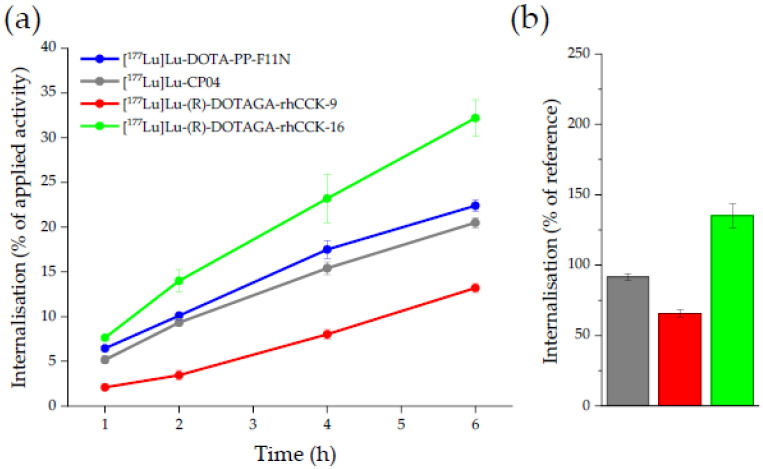
(**a**) CCK-2R-mediated internalisation (0.25 pmol/well) on AR42J cells as percent (%) of the applied activity (incubation at 37 °C for 1, 2, 4 and 6 h, RPMI 1640, 5 mm L-Gln, 5 mL non-essential amino acids (100×), 10% FCS + 5% BSA (*v*/*v*), 3.0 × 10^5^ cells/mL/well). (**b**) CCK-2R mediated internalisation (% of the reference [^177^Lu]Lu-DOTA-PP-F11N) of [^177^Lu]Lu-CP04 (grey), [^177^Lu]Lu-(*R*)-DOTAGA-rhCCK-9 (red) and [^177^Lu]Lu-(*R*)-DOTAGA-rhCCK-16 (green) after incubation for 6 h.

Internalisation values at different time points were determined for the respective most affine *α*- and *γ*-linked rhCCK derivative ([^177^Lu]Lu-(*R*)-DOTAGA-rhCCK-9 and -16) compared to the references. The amount of internalised activity (%) on AR42J cells increased over time for all compounds tested from 2–8% (1 h) to 13–32% (6 h) (Figure 3, Table S2). Most of the cell-associated activity was internalized, while cell membrane-bound activity was ≤1.1% (Tables S2 and S3).

The ^177^Lu-labelled rhCCK derivative [^177^Lu]Lu-(*R*)-DOTAGA-rhCCK-16 exhibited distinctly higher internalisation values than the reference compounds, [^177^Lu]Lu-DOTA-PP-F11N and [^177^Lu]Lu-CP04. In comparison, the internalisation kinetics of [^177^Lu]Lu-(*R*)-DOTAGA-rhCCK-9 were found to be lower.

### 2.3. In Vivo Characterisation

**Figure 4:**
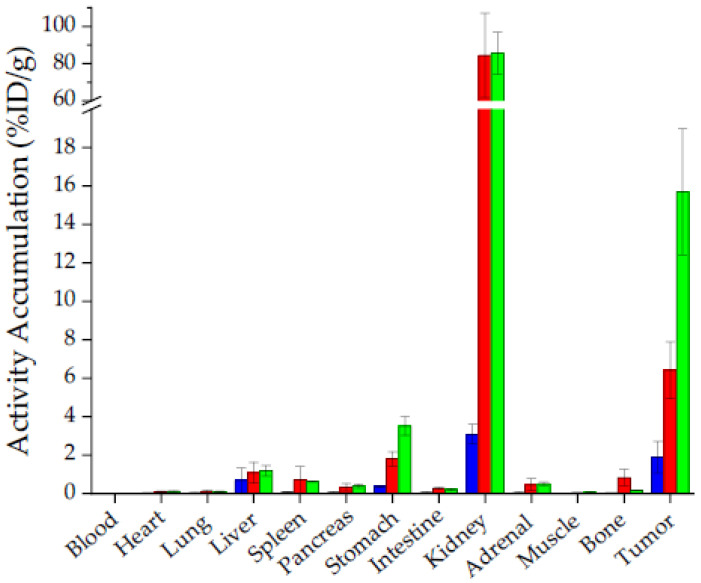
Biodistribution of the reference compound, [^177^Lu]Lu-DOTA-PP-F11N (blue), the *α*-linked [^177^Lu]Lu-(*R*)-DOTAGA-rhCCK-9 (red) and the *γ*-linked [^177^Lu]Lu-(*R*)-DOTAGA-rhCCK-16 (green) in selected organs (%ID/g) at 24 h p.i. in AR42J tumour-bearing CB17-SCID mice (100 pmol each, *n* = 4). Data is expressed as mean ± SD.

The most affine *α*-linked([^177^Lu]Lu-(*R*)-DOTAGA-rhCCK-9) and *γ*-linked ([^177^Lu]Lu-(*R*)-DOTAGA-rhCCK-16) rhCCK derivatives were evaluated in vivo in comparison to the reference [^177^Lu]Lu-DOTA-PP-F11N, which is already being applied in clinical trials (Figure 4, Table S4).

In vivo, the rhCCK derivatives showed 3- (6.40 ± 1.48 %ID/g, [^177^Lu]Lu-(*R*)-DOTAGA-rhCCK-9) to 8-fold (15.7 ± 3.3 %ID/g, [^177^Lu]Lu-(*R*)-DOTAGA-rhCCK-16) higher activity levels in the tumour and 5- to 10-fold higher levels in the CCK-2R-positive stomach than the reference ligand, which was statistically significant in groups of four mice (*p* < 0.02). Activity levels in the liver were not significantly increased for both rhCCK derivatives compared to the reference compound at 24 h p.i. despite their increased lipophilicity (*p* > 0.15). Blood levels of both rhCCK derivatives were 10- to 11-fold increased to [^177^Lu]Lu-DOTA-PP-F11N (*p* < 0.001) but still favourably low at 24 h p.i. (~0.015 %ID/g) despite their decelerated clearance kinetics. However, activity levels in the kidneys were almost 30-fold higher for the rhCCK derivatives compared to [^177^Lu]Lu-DOTA-PP-F11N at 24 h p.i. (84.4 ± 22.7 and 85.5 ± 11.3 vs. 3.08 ± 0.51 %ID/g, respectively, *p* < 0.001). [^177^Lu]Lu-DOTA-PP-F11N revealed lower activity levels in most organs at 24 h p.i., indicating a more rapid clearance.

**Figure 5:**
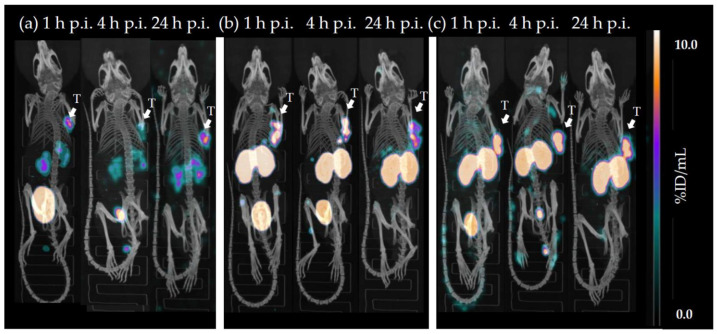
Representative *µ*SPECT/CT images of (**a**) [^177^Lu]Lu-DOTA-PP-F11N, (**b**) [^177^Lu]Lu-*(R)-*DOTAGA-rhCCK-9 and (**c**) [^177^Lu]Lu-*(R)-*DOTAGA-rhCCK-16 at 1, 4 and 24 h p.i. in AR42J tumour-bearing CB17- SCID mice (100 pmol each). Tumours (T) are indicated by white arrows.

*µ*SPECT/CT studies of mice (*n* = 1) injected with [^177^Lu]Lu-(*R*)-DOTAGA-rhCCK-9, [^177^Lu]Lu-(*R*)-DOTAGA-rhCCK-16 and [^177^Lu]Lu-DOTA-PP-F11N at 1, 4 and 24 h p.i. revealed a low overall background activity for all three compounds at each time point, except for a high kidney accumulation and retention for the latter two (Figure 5). Activity levels in the tumour were highest for [^177^Lu]Lu-(*R*)-DOTAGA-rhCCK-16 (15.7 ± 3.3 %ID/g) and lowest for the reference ligand (1.8 ± 0.8 %ID/g).

CCK-2R specificity of [^177^Lu]Lu-(*R*)-DOTAGA-rhCCK-16 was evaluated via co-injection of an excess of the CCK2R-targeted compound, [^nat^Lu]Lu-DOTA-MGS5, which resulted in activity levels in the tumour < 1%. Activity levels in the stomach, which endogenously expresses the CCK-2R, were found to be <0.3% (Figure S4, Table S4).

## 3. Discussion

Among the currently most promising minigastrin-derived peptides for clinical application (DOTA-MGS5, DOTA-PP-F11N and CP04), there is no option available for facile ^18^F-labelling [20,21,22,23]. Particularly a ^18^F-labelled minigastrin analogue would be beneficial for the detection of small MTC-derived metastases due to the low tissue penetration and thus high resolution of fluorine-18, consequently to its low positron energy (*E*_β,max_ = 635 keV), compared to gallium-68, for example [24]. Furthermore, up to now there is no CCK-2R-targeted ligand that enables both ^18^F- and ^177^Lu-labelling. This radiohybrid-based concept has been successfully implemented for PSMA inhibitors and resulted in impressive results over the last three years, which is why the compounds rhPSMA-7.3 and rhPSMA-10.1 are evaluated in clinical studies [13,25,26,27,28,29,30,31,32]. With the aim to develop a ^177^Lu-labelled minigastrin analogue, which can also be labelled with fluorine-18, we introduced the lipophilic SiFA moiety into different sites within the highly hydrophilic, *N*-terminal D-glutamate chain of DOTA-PP-F11N and compared these novel compounds to the reference compounds, [^177^Lu]Lu-DOTA-PP-F11N and [^177^Lu]Lu-CP04.

The SiFA moiety was introduced into different sites within both an *α*-linked poly-D-glutamate chain (= (*R*)-DOTAGA-rhCCK-1 to -9) and a *γ*-linked poly-D-glutamate chain (= (*R*)-DOTAGA-rhCCK-10 to -18), while the binding sequence of DOTA-PP-F11N was maintained. In general, for both linker concepts it could be observed that with increasing distance of the SiFA moiety to the binding motif, CCK-2R affinity was enhanced, which indicates that the bulky SiFA building block does not fit into the binding pocket of the receptor. However, at a farther distance the SiFA group seems to be located outside of the binding pocket and thus CCK-2R affinity increases. In direct comparison of these series of minigastrin derivatives (*α*- or *γ*-linked D-glutamate chain) that each exhibit the same site for the SiFA moiety, it is evident that those ligands containing a *γ*-linked D-glutamate chain generally show a higher CCK-2R affinity. Similar results were observed for the respective *γ*-linked analogues of the references, [^nat^Lu]Lu-DOTA-PP-*γ*-F11N and [^nat^Lu]Lu-*γ*-CP04, pointing to a beneficial effect of a prolonged linker section and thus the use of *γ*-linked D-glutamate residues. Nevertheless, *IC*_50_ values of the most affine compounds were still approximately fivefold (*α*-linked poly-D-glutamate linker) and twofold (*γ*-linked poly-D-glutamate linker) higher compared to [^nat^Lu]Lu-DOTA-PP-F11N and [^nat^Lu]Lu-CP04 (*IC*_50_ of 11–13 nM). Hence, it is assumed that the lower CCK-2R affinity of the novel rhCCK derivatives is due either to the addition of the sterically demanding SiFA moiety or the (*R*)-DOTAGA chelator, which comprises one negative charge more than the DOTA chelator present in the reference compounds when labelled with [^nat/177^Lu]lutetium.

The addition of the SiFA moiety was also accompanied by an enhanced lipophilicity (log*D*_7.4_: −2.9 to −1.7), which is one to three magnitudes higher compared to [^177^Lu]Lu-DOTA-PP-F11N and [^177^Lu]Lu-CP04. However, this was desired since for tumour targeting we consider a log*D*_7.4_ value of about −4 or lower unfavourable because we assume that high tumour uptake is prevented by a too rapid clearance rate. Indeed, first patient studies with [^177^Lu]Lu-DOTA-PP-F11N showed low activity levels in the tumour but high levels in the bladder at 1 h p.i., most likely due to an accelerated clearance [12]. Based on previous experiences in our group, a range of −3 to −2 seems to be ideal for tumour targeting.

**Figure 6:**
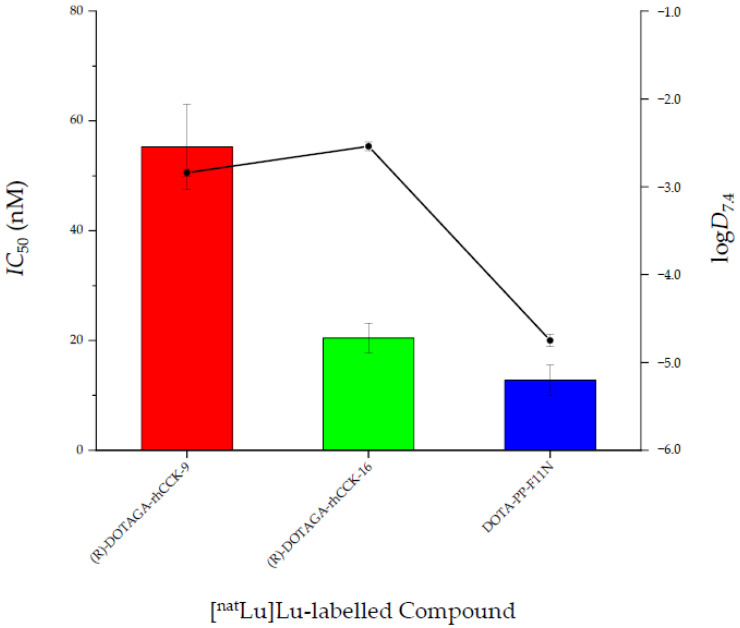
Affinity (*IC*_50_) and lipophilicity (log*D*_7.4_) data of ^nat/177^Lu-labelled rhCCK ligands, (*R*)-DOTAGA-rhCCK-9 and -16, as well as DOTA-PP-F11N.

We thus selected the most promising *α*- and *γ*-linked compound with regard to CCK-2R affinity (*IC*_50_) and lipophilicity (log*D*_7.4_), (*R*)-DOTAGA-rhCCK-9 and -16 (Figure 6), respectively, for in vivo studies.

Interestingly, both SiFA-containing compounds revealed noticeably higher activity levels in the tumour at all time points than [^177^Lu]Lu-DOTA-PP-F11N despite their distinctly lower CCK-2R affinity. [^177^Lu]Lu-(*R*)-DOTAGA-rhCCK-16 revealed a higher CCK-2R-mediated internalisation than [^177^Lu]Lu-DOTA-PP-F11N, which points to an enhanced uptake by the tumour cells. [^177^Lu]Lu-(*R*)-DOTAGA-rhCCK-9 revealed low internalisation values, which is in accordance with its low CCK-2R affinity but contradicting to the higher tumour uptake found compared to the reference. It has to be added that tumour values for [^177^Lu]Lu-DOTA-PP-F11N were low despite its high CCK-2R affinity, which is, however, in accordance to the patient data and most likely caused its high hydrophilicity and thus rapid clearance [12]. Although further studies have to be carried out to elucidate the beneficial effects observed in this study, we proved that the introduction of the SiFA group not only generated a possibility for ^18^F-labelling but also improved overall bioavailability in vivo. Besides the decreased hydrophilicity, we further suspect an elevated albumin binding potential of the SiFA group, which decelerates the activity clearance, increases circulation time of the compounds in the blood and thus enhances activity uptake and retention in the tumour. Similar observations were made for PSMA-targeted compounds [14,33].

Hence, [^177^Lu]Lu-(*R*)-DOTAGA-rhCCK-9 and -16 showed 3- and 8-fold higher activity levels in the tumour, respectively, than the reference ligand at 24 h p.i. Similar observations were made for stomach levels due to the endogenous CCK-2R expression in this organ. Competition studies using an excess of the CCK-2R-specific ligand, [^nat^Lu]Lu-DOTA-MGS5 [34], confirmed CCK-2R specificity. Despite their enhanced lipophilicity, liver levels were not significantly increased compared to [^177^Lu]Lu-DOTA-PP-F11N at 24 h p.i. Blood levels of both rhCCK derivatives were significantly higher than those of [^177^Lu]Lu-DOTA-PP-F11N but still in a comparable range to compounds addressing other tumour targets in nuclear medicine, such as PSMA-, gastrin releasing peptide receptor-, chemokine receptor CXCR4- and somatostatin-2 receptor-targeted probes [14,19,35,36,37].

Despite these respectable results, our current rhCCK derivatives suffer from elevated activity levels in the kidneys (30-fold higher compared to the reference). We assume a synergistic effect of the negative charges in proximity of the SiFA moiety within the linker section, as [^177^Lu]Lu-DOTA-PP-F11N did not show comparable kidney values although it comprises a similar amount of negative charges in its linker. These kidney issues have to be addressed in future studies to enable a clinical translation of this rh-based concept for minigastrin analogues. One possible strategy to decrease kidney accumulation and retention might be a reduction of the albumin binding of the rhCCK derivatives, as beneficial effects were observed for PSMA inhibitors when negative charges in direct proximity to the SiFA-building block were depleted [14]. Furthermore, co-injection of lysine or gelofusine could also be a valuable tool. Tumour values were noticeably higher at 1 and 4 h p.i. compared to the reference in *µ*SPECT/CT imaging (*n* = 1). However, further studies at 1 and 4 h p.i. have to be conducted to statistically confirm these observations and, furthermore, elucidate the imaging potential of the respective ^18^F-labeled rhCCK analogues.

In summary, we could successfully introduce a SiFA building block into the minigastrin analogue DOTA-PP-F11N, which not only generated a possibility for ^18^F-labelling but also considerably improved pharmacokinetics. We further could show that the rh-based concept successfully applied for PSMA-targeted compounds can be applied for CCK-2R-targeted ligands as well, which enables both ^18^F- and ^177^Lu-labelling for a theranostic use. Nevertheless, elevated activity levels in the kidneys are of concern, which has to be optimised in future studies. Moreover, CCK-2R affinity might possibly be further improved, either by varying the position of the SiFA building block or a DOTA-for-(*R*)-DOTAGA substitution. However, a beneficial effect of a *γ*- instead of an *α*-linked D-glutamate chain in minigastrin derivatives was found, which might be applicable for other peptides and their linker as well.

## 4. Materials and Methods

Characterisation of all CCK-2R-targeted compounds is provided in the Supplementary Materials (Figures S1–S4). Electrospray ionisation-mass spectra for characterisation of the substances were acquired on an expression^L^ CMS mass spectrometer (Advion Ltd., Harlow, UK).

### 4.1. Chemical Synthesis and Labelling Procedures

All compounds were synthesised via standard Fmoc-based solid phase peptide synthesis (SPPS) using a H-Rink Amide ChemMatrix^®^ resin (35–100 mesh particle size, 0.4–0.6 mmol/g loading, Merck KGaA, Darmstadt, Germany). Final purification of the peptides was performed by reversed phase high performance liquid chromatography (RP-HPLC).

^177^Lu- and ^nat^Lu-complexation of the peptides was performed according to a previously published procedure [14].

### 4.2. In Vitro Experiments

Detailed description of all cell-based experiments is provided in the Supplementary Materials. In brief, competitive binding studies were conducted on AR42J cells (2.0 × 10^5^ cells per 1 mL/well) via incubation at 37 °C for 3 h using [^177^Lu]Lu-DOTA-PP-F11N (0.3 pmol) as a radiolabelled reference (*n* = 3).

Internalisation studies of the ^177^Lu-labelled conjugates (0.3 pmol) were performed on AR42J cells (3.0 × 10^5^ cells per 1 mL/well) at 37 °C for 1, 2, 4 and 6 h (*n* = 3). Data were corrected for non-specific binding (competition by 10^−4^ M [^nat^Lu]Lu-DOTA-PP-F11N).

Lipophilicity (depicted as octanol-phosphate-buffered saline solution (PBS, pH = 7.4) distribution coefficient, log*D*_7.4_) was determined via dissolving the ^177^Lu-labelled peptide (approx. 1 MBq) in a mixture (1/1, *v*/*v*) of n-octanol and PBS. The suspension was vortexed in a reaction vial (1.5 mL) for 3 min at RT and the vial was centrifuged at 9000× *g* rpm for 5 min (Biofuge 15, Heraus Sepatech GmbH, Osterode, Germany). 200 μL aliquots of both layers were measured separately in a *γ*-counter (Perkin Elmer, Waltham, MA, USA). The experiment was repeated at least five times.

### 4.3. In Vivo Experiments

All animal experiments were conducted in accordance with general animal welfare regulations in Germany (German animal protection act, in the edition of the announcement, dated 18 May 2006, as amended by Article 280 of 19 June 2020, approval no. ROB-55.2-1-2532.Vet_02-18-109 by the General Administration of Upper Bavaria) and the institutional guidelines for the care and use of animals. CB17-SCID mice of both genders and aged 2–12 months (Charles River Laboratories International Inc., Sulzfeld, Germany) were allowed to acclimate at the in-house animal facility for at least one week prior to tumour cell inoculation was performed. Tumour xenografts were generated using AR42J cells (5.0 × 10^6^ cells per 200 µL) suspended in a 1/1 mixture (*v*/*v*) of RPMI 1640 medium and Cultrex^®^ Basement Membrane Matrix Type 3 (Trevigen, Gaithersburg, MD, USA). This suspension was inoculated subcutaneously onto the right shoulder and animals were used when tumour volume was >100 mm^3^ (1–2 week after inoculation). Exclusion criteria for animals from an experiment were either weight loss higher than 20%, a tumour size above 1500 mm^3^, an ulceration of the tumour, respiratory distress or a change of behaviour. None of these criteria applied to any animal from the experiment. Neither randomisation nor blinding was applied in the allocation of the experiments. Health status is SPF according to FELASA.

For biodistribution studies, the ^177^Lu-labelled compound (approx. 2–3 MBq, 100 pmol) was injected into a lateral tail vein (*n* = 4) of anesthetised (2% isoflurane) AR42J tumour-bearing CB-17-SCID mice. At 24 h post-injection (p.i.), the mice were euthanised. Thereafter, the pertinent organs were removed, weighed and measured using a *γ*-counter.

Imaging studies were carried out according to a recently published protocol [19]. Static images were recorded at t = 1, 4 and 24 h p.i. (anesthesia by 2% isoflurane, *n* = 1) with an acquisition time of t + (45–60 min) using a high-energy general-purpose rat and mouse collimator via MILabs acquisition software v11.00 and v12.26 from MILabs (Utrecht, The Netherlands).

For all competition studies, 2.90 mg/kg (40 nmol) of [^nat^Lu]Lu-DOTA-MGS5 (10^−3^ M in phosphate-buffered saline) were co-administered.

Acquired data were statistically analysed by performing a Student’s t-test via Excel (Microsoft Corporation, Redmond, WA, USA) and OriginPro software (version 9.7) from OriginLab Corporation (Northampton, MA, USA). Acquired *p* values of <0.05 were considered statistically significant.

## 5. Conclusions

We could demonstrate that the radiohybrid-based concept could easily be transferred to minigastrin derivatives, whose hydrophilic linker section compensates for the high lipophilicity of the introduced SiFA moiety. This offers not only the possibility of ^18^F- and ^177^Lu-labelling with the same molecule but also had a beneficial impact on overall pharmacokinetics, as clearance kinetics were decelerated. Thereby, activity retention in the tumour could be increased by approximately eightfold compared to the clinically applied [^177^Lu]Lu-DOTA-PP-F11N. However, these compounds also suffer from a noticeably enhanced kidney retention. This will be addressed in further studies.

## Data Availability

Data is contained within the article and Supplementary Materials.

## Supplementary Materials

The following supporting information can be downloaded at: https://www.mdpi.com/article/10.3390/ph15121467/s1, general information and characterisation of all CCK2R-targeted compounds, detailed description of cell-based experiments. Figure S1: Confirmation of peptide identity and integrity for (**a**) [^nat^Lu]Lu-DOTA-PP-F11N and (**b**) [^177^Lu]Lu-DOTA-PP-F11N as analysed by analytical (radio-)RP-HPLC (MultoKrom 100-5 C18, 5 μm, 125 × 4.6 mm, CS Chromatographie GmbH, Langerwehe, Germany; 10→90% MeCN in H_2_O + 0.1% TFA in 15 min). (**c**) Mass spectrum of [^nat^Lu]Lu-DOTA-PP-F11N; Figure S2: Confirmation of peptide identity and integrity for (**a**) [^nat^Lu]Lu-(*R*)-DOTAGA-rhCCK-9 and (**b**) [^177^Lu]Lu-(*R*)-DOTAGA-rhCCK-9 as analysed by analytical (radio-)RP-HPLC (MultoKrom 100-5 C18, 5 μm, 125 × 4.6 mm, CS Chromatographie GmbH, Langerwehe, Germany; 10→90% MeCN in H_2_O + 0.1% TFA in 15 min). (**c**) Mass spectrum of [^nat^Lu]Lu-(*R*)-DOTAGA-rhCCK-9; Figure S3: Confirmation of peptide identity and integrity for (**a**) [^nat^Lu]Lu-(*R*)-DOTAGA-rhCCK-16 and (**b**) [^177^Lu]Lu-(*R*)-DOTAGA-rhCCK-16 as analysed by analytical (radio-)RP-HPLC (MultoKrom 100-5 C18, 5 μm, 125 × 4.6 mm, CS Chromatographie GmbH, Langerwehe, Germany; 10→90% MeCN in H_2_O + 0.1% TFA in 15 min). (**c**) Mass spectrum of [^nat^Lu]Lu-(*R*)-DOTAGA-rhCCK-16; Figure S4: (**a**) Biodistribution of [^177^Lu]Lu-DOTA-rhCCK-16 (100 pmol) co-injected with [^nat^Lu]Lu-DOTA-MGS5 (40 nmol) in selected organs (%ID/g) at 24 h p.i. in AR42J tumour-bearing CB17-SCID mice. Data is expressed as mean ± SD (*n* = 2). (**b**) Representative *µ*SPECT/CT images of [^177^Lu]Lu-DOTA-rhCCK-16 co-injected with [^nat^Lu]Lu-DOTA-MGS5 (40 nmol) at 24 h p.i. in AR42J tumour-bearing CB17- SCID mice; Table S1: Affinity and lipophilicity data of the compounds evaluated. Affinity data were determined on AR42J cells (2.0 × 10^5^ cells/well) and [^177^Lu]Lu-DOTA-PP-F11N (0.3 pmol/well) as radiolabelled reference (3 h, 37 °C, RPMI 1640, 5 mM L-Gln, 5 mL non-essential amino acids (100×), 10% FCS + 5% BSA (*v*/*v*)); Table S2: Receptor-mediated internalisation values (37 °C, RPMI 1640, 5 mM L-Gln, 5 mL non-essential amino acids (100×), 10% FCS, 0.25 pmol/well) determined as percent (%) of the applied activity of [^177^Lu]Lu-(*R*)-DOTAGA-rhCCK-16 and the references [^177^Lu]Lu-DOTA-PP-F11N and [^177^Lu]Lu-CP04 using AR42J cells (3.0 × 10^5^ cells/well) at different time points (1, 2, 4 and 6 h). Data are corrected for non-specific binding (10 µmol/well, [^nat^Lu]Lu-DOTA-PP-F11N); Table S3: Total cell uptake (37 °C, RPMI 1640, 5 mM L-Gln, 5 mL non-essential amino acids (100×), 10% FCS, 0.25 pmol/well) determined as percent (%) of the applied activity of [^177^Lu]Lu-(*R*)-DOTAGA-rhCCK-9 and -16 as well as the references [^177^Lu]Lu-DOTA-PP-F11N and [^177^Lu]Lu-CP04 using AR42J cells (3.0 × 10^5^ cells/well) at different time points (1, 2, 4 and 6 h). Data are corrected for non-specific binding (10 µmol/well, [^nat^Lu]Lu-DOTA-PP-F11N); Table S4: Biodistribution data of [^177^Lu]Lu-DOTA-PP-F11N, [^177^Lu]Lu-(*R*)-DOTAGA-rhCCK-9 and [^177^Lu]Lu-(*R*)-DOTAGA-rhCCK-16 in selected organs at 24 h p.i. in AR42J tumour-bearing CB17-SCID mice (100 pmol each). Data are expressed as %ID/g, mean ± SD.

## References

[1] Stamatakos M., Paraskeva P., Stefanaki C., Katsaronis P., Lazaris A., Safioleas K., Kontzoglou K. (2011). Medullary thyroid carcinoma: The third most common thyroid cancer reviewed. Oncol. Lett..

[2] Wells S.A., Asa S.L., Dralle H., Elisei R., Evans D.B., Gagel R.F., Lee N., Machens A., Moley J.F., Pacini F. (2015). Revised American Thyroid Association guidelines for the management of medullary thyroid carcinoma. Thyroid.

[3] Hadoux J., Schlumberger M. (2017). Chemotherapy and tyrosine-kinase inhibitors for medullary thyroid cancer. Best Pract. Res. Clin. Endocrinol. Metab..

[4] Hazard J.B. (1977). The C cells (parafollicular cells) of the thyroid gland and medullary thyroid carcinoma. A review. Am. J. Pathol..

[5] Resteghini C., Cavalieri S., Galbiati D., Granata R., Alfieri S., Bergamini C., Bossi P., Licitra L., Locati L.D. (2017). Management of tyrosine kinase inhibitors (TKI) side effects in differentiated and medullary thyroid cancer patients. Best Pract. Res. Clin. Endocrinol. Metab..

[6] Reubi J.C., Waser B. (1996). Unexpected high incidence of cholecystokinin-B/gastrin receptors in human medullary thyroid carcinomas. Int. J. Cancer.

[7] Behr T.M., Jenner N., Béhé M., Angerstein C., Gratz S., Raue F., Becker W. (1999). Radiolabeled Peptides for Targeting Cholecystokinin-B/Gastrin Receptor-Expressing Tumors. J. Nucl. Med..

[8] Behe M., Becker W., Gotthardt M., Angerstein C., Behr T.M. (2003). Improved kinetic stability of DTPA-dGlu as compared with conventional monofunctional DTPA in chelating indium and yttrium: Preclinical and initial clinical evaluation of radiometal labelled minigastrin derivatives. Eur. J. Nucl. Med. Mol. Imaging.

[9] Laverman P., Joosten L., Eek A., Roosenburg S., Peitl P.K., Maina T., Macke H., Aloj L., von Guggenberg E., Sosabowski J.K. (2011). Comparative biodistribution of 12 (1)(1)(1)In-labelled gastrin/CCK2 receptor-targeting peptides. Eur. J. Nucl. Med. Mol. Imaging.

[10] Sauter A.W., Mansi R., Hassiepen U., Muller L., Panigada T., Wiehr S., Wild A.M., Geistlich S., Béhé M., Rottenburger C. (2019). Targeting of the Cholecystokinin-2 Receptor with the Minigastrin Analog (177)Lu-DOTA-PP-F11N: Does the Use of Protease Inhibitors Further Improve In Vivo Distribution?. J. Nucl. Med..

[11] Martin B., Schibli R. (2018). Mini-Gastrin Analogue, in Particular for Use in CCK2 Receptor Positive Tumour Diagnosis and/or Treatment. U.S. Patent.

[12] Rottenburger C., Nicolas G.P., McDougall L., Kaul F., Cachovan M., Vija A.H., Schibli R., Geistlich S., Schumann A., Rau T. (2020). Cholecystokinin 2 Receptor Agonist (177)Lu-PP-F11N for Radionuclide Therapy of Medullary Thyroid Carcinoma: Results of the Lumed Phase 0a Study. J. Nucl. Med..

[13] Wurzer A., Di Carlo D., Schmidt A., Beck R., Eiber M., Schwaiger M., Wester H.-J. (2020). Radiohybrid Ligands: A Novel Tracer Concept Exemplified by 18F- or 68Ga-Labeled rhPSMA Inhibitors. J. Nucl. Med..

[14] Wurzer A., Kunert J.P., Fischer S., Felber V., Beck R., Rose F., D’Alessandria C., Weber W., Wester H.J. (2022). Synthesis and Preclinical Evaluation of (177)Lu-Labeled Radiohybrid PSMA Ligands for Endoradiotherapy of Prostate Cancer. J. Nucl. Med..

[15] Eiber M., Kronke M., Wurzer A., Ulbrich L., Jooss L., Maurer T., Horn T., Schiller K., Langbein T., Buschner G. (2020). (18)F-rhPSMA-7 positron emission tomography for the detection of biochemical recurrence of prostate cancer following radical prostatectomy. J. Nucl. Med..

[16] Kronke M., Wurzer A., Schwamborn K., Ulbrich L., Jooss L., Maurer T., Horn T., Rauscher I., Haller B., Herz M. (2020). Histologically-confirmed diagnostic efficacy of (18)F-rhPSMA-7 positron emission tomography for N-staging of patients with primary high risk prostate cancer. J. Nucl. Med..

[17] Oh S.W., Wurzer A., Teoh E.J., Oh S., Langbein T., Kronke M., Herz M., Kropf S., Wester H.J., Weber W.A. (2020). Quantitative and Qualitative Analyses of Biodistribution and PET Image Quality of Novel Radiohybrid PSMA, (18)F-rhPSMA-7, in Patients with Prostate Cancer. J. Nucl. Med..

[18] Kroenke M., Mirzoyan L., Horn T., Peeken J.C., Wurzer A., Wester H.J., Makowski M., Weber W.A., Eiber M., Rauscher I. (2021). Matched-Pair Comparison of (68)Ga-PSMA-11 and (18)F-rhPSMA-7 PET/CT in Patients with Primary and Biochemical Recurrence of Prostate Cancer: Frequency of Non-Tumor-Related Uptake and Tumor Positivity. J. Nucl. Med..

[19] Guenther T., Deiser S., Felber V., Beck R., Wester H.J. (2022). Substitution of L-Tryptophan by a-Methyl-L-Tryptophan in 177Lu-RM2 Results in 177Lu-AMTG, a High-Affinity Gastrin-Releasing Peptide Receptor Ligand with Improved In Vivo Stability. J. Nucl. Med..

[20] Uprimny C., von Guggenberg E., Svirydenka A., Mikolajczak R., Hubalewska-Dydejczyk A., Virgolini I.J. (2020). Comparison of PET/CT imaging with [(18)F]FDOPA and cholecystokinin-2 receptor targeting [(68)Ga]Ga-DOTA-MGS5 in a patient with advanced medullary thyroid carcinoma. Eur. J. Nucl. Med. Mol. Imaging.

[21] Hörmann A.A., Klingler M., Rangger C., Mair C., Decristoforo C., Uprimny C., Virgolini I.J., von Guggenberg E. (2021). Radiopharmaceutical Formulation and Preclinical Testing of 68Ga-Labeled DOTA-MGS5 for the Regulatory Approval of a First Exploratory Clinical Trial. Pharmaceuticals.

[22] 177Lu-PP-F11N for Receptor Targeted Therapy and Imaging of Metastatic Thyroid Cancer. https://ClinicalTrials.gov/show/NCT02088645.

[23] Radiolabelled CCK-2/Gastrin Receptor Analogue for Personalized Theranostic Strategy in Advanced MTC. https://ClinicalTrials.gov/show/NCT03246659.

[24] Bernard-Gauthier V., Wangler C., Schirrmacher E., Kostikov A., Jurkschat K., Wangler B., Schirrmacher R. (2014). (1)(8)F-labeled silicon-based fluoride acceptors: Potential opportunities for novel positron emitting radiopharmaceuticals. Biomed. Res. Int..

[25] Malaspina S., Taimen P., Kallajoki M., Oikonen V., Kuisma A., Ettala O., Mattila K., Boström P.J., Minn H., Kalliokoski K. (2022). Uptake of (18)F-rhPSMA-7.3 in Positron Emission Tomography Imaging of Prostate Cancer: A Phase 1 Proof-of-Concept Study. Cancer Biother. Radiopharm..

[26] Feuerecker B., Chantadisai M., Allmann A., Tauber R., Allmann J., Steinhelfer L., Rauscher I., Wurzer A., Wester H.J., Weber W.A. (2021). Pre-therapeutic comparative dosimetry of (177)Lu-rhPSMA-7.3 and (177)Lu-PSMAI&T in patients with metastatic castration resistant prostate cancer (mCRPC). J. Nucl. Med..

[27] Yusufi N., Wurzer A., Herz M., D’Alessandria C., Feuerecker B., Weber W., Wester H.J., Nekolla S., Eiber M. (2021). Comparative Preclinical Biodistribution, Dosimetry, and Endoradiotherapy in Metastatic Castration-Resistant Prostate Cancer Using (19)F/(177)Lu-rhPSMA-7.3 and (177)Lu-PSMA I&T. J. Nucl. Med..

[28] Imaging Study to Investigate the Safety and Diagnostic Performance of rhPSMA 7.3 (18F) in Newly Diagnosed Prostate Cancer (LIGHTHOUSE). https://ClinicalTrials.gov/show/NCT04186819.

[29] Imaging Study to Investigate Safety and Diagnostic Performance of rhPSMA 7.3 (18F) PET Ligand in Suspected Prostate Cancer Recurrence (SPOTLIGHT). https://ClinicalTrials.gov/show/NCT04186845.

[30] Anti-tumour Activity of (177Lu) rhPSMA-10.1 Injection. https://ClinicalTrials.gov/show/NCT05413850.

[31] Assessing Radio-hybrid Prostate Specific Membrane Antigen (rhPSMA-7.3) (18F) in Healthy Volunteers and Subjects with Prostate Cancer. https://ClinicalTrials.gov/show/NCT03995888.

[32] An Investigational Scan (rh PSMA 7.3 PET/MRI) for the Detection of Recurrent Disease and Aid in Radiotherapy Planning in Biochemically Recurrent Prostate Cancer. https://ClinicalTrials.gov/show/NCT04978675.

[33] Kunert J.P., Fischer S., Wurzer A., Wester H.J. (2022). Albumin-Mediated Size Exclusion Chromatography: The Apparent Molecular Weight of PSMA Radioligands as Novel Parameter to Estimate Their Blood Clearance Kinetics. Pharmaceuticals.

[34] Klingler M., Summer D., Rangger C., Haubner R., Foster J., Sosabowski J., Decristoforo C., Virgolini I., von Guggenberg E. (2019). DOTA-MGS5, a New Cholecystokinin-2 Receptor-Targeting Peptide Analog with an Optimized Targeting Profile for Theranostic Use. J. Nucl. Med..

[35] Schottelius M., Osl T., Poschenrieder A., Hoffmann F., Beykan S., Hänscheid H., Schirbel A., Buck A.K., Kropf S., Schwaiger M. (2017). [(177)Lu]pentixather: Comprehensive Preclinical Characterization of a First CXCR4-directed Endoradiotherapeutic Agent. Theranostics.

[36] Dalm S.U., Nonnekens J., Doeswijk G.N., de Blois E., van Gent D.C., Konijnenberg M.W., de Jong M. (2016). Comparison of the Therapeutic Response to Treatment with a 177Lu-Labeled Somatostatin Receptor Agonist and Antagonist in Preclinical Models. J. Nucl. Med..

[37] Nicolas G.P., Mansi R., McDougall L., Kaufmann J., Bouterfa H., Wild D., Fani M. (2017). Biodistribution, Pharmacokinetics, and Dosimetry of (177)Lu-, (90)Y-, and (111)In-Labeled Somatostatin Receptor Antagonist OPS201 in Comparison to the Agonist (177)Lu-DOTATATE: The Mass Effect. J. Nucl. Med..

