# Supplementary material for: Introduction of a SiFA Moiety into the D-Glutamate Chain of DOTA-PP-F11N Results in Radiohybrid-Based CCK-2R-Targeted Compounds with Improved Pharmacokinetics In Vivo
Source: Pharmaceuticals (Basel). 2022 Nov 25;15(12):1467. doi: 10.3390/ph15121467 (PMC9783573; doi:10.3390/ph15121467)
Supplement: Supplementary file 1 [file pharmaceuticals-15-01467-s001.zip › pharmaceuticals-2050466-supplementary.pdf]

Introduction of a SiFA Moiety into the D-Glutamate Chain of DOTA-PP-F11N Results in Radiohybrid-Based CCK-2R-Targeted Compounds with Improved Pharmacokinetics *in vivo*

# - Supplementary Materials -

Nadine Holzleitner[1,#,*] Thomas Günther[1,#], Roswitha Beck[1], Constantin Lapa[2] and Hans-Jürgen Wester[1]

[1]Chair of Pharmaceutical Radiochemistry, Department of Chemistry, Technical University of Munich, Garching, Germany

[2]Clinic for Nuclear Medicine, University Hospital Augsburg, Augsburg, Germany

Corresponding co-authors:

Nadine Holzleitner and Thomas Günther

Phone: +49.89.289.12203

Technical University of Munich,

Chair of Pharmaceutical Radiochemistry,

Walther-Meissner-Str. 3

85748 Garching

GERMANY

Fax: +49.89.289.12204

E-Mail: nadine.holzleitner@tum.de and thomas.guenther@tum.de

ORCID: https://orcid.org/0000-0001-8258-3526 (NH) and https://orcid.org/0000-0002-7412-0297 (TG)

Analytical data of $^{nat/177}$Lu-labeled minigastrin analogues

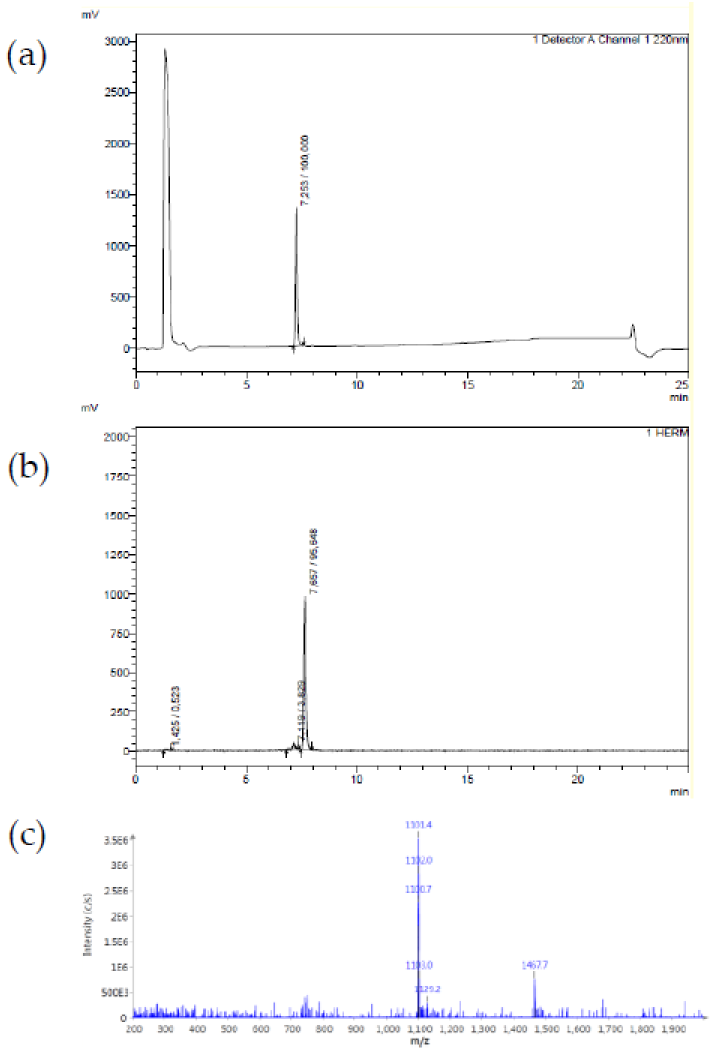

Figure S1. Confirmation of peptide identity and integrity for (**a**) [^{nat}Lu]Lu-DOTA-PP-F11N and (**b**) [^{177}Lu]Lu-DOTA-PP-F11N as analysed by analytical (radio-)RP-HPLC (MultoKrom 100-5 C18, 5 µm, 125 × 4.6 mm, CS Chromatographie GmbH, Langerwehe, Germany; 10→90% MeCN in H_2O + 0.1% TFA in 15 min). (**c**) Mass spectrum of [^{nat}Lu]Lu-DOTA-PP-F11N.

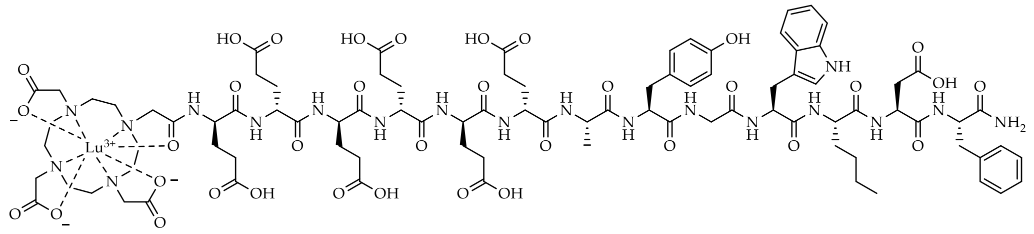

[natLu]Lu-DOTA-PP-F11N: RP-HPLC (10→90% MeCN in H_2O with 0.1% TFA, 15 min, $\lambda = 220$ nm): $t_R = 7.55$ min, $K' = 3.48$; MS (ESI, positive): m/z calculated for $C_{90}H_{120}LuN_{19}O_{35}$: 2203.0, found: m/z = 1101.4 $[M+2H]^{2+}$.

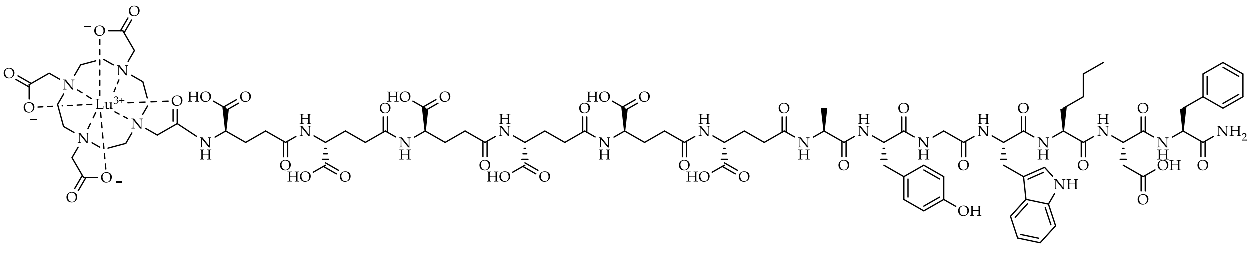

[natLu]Lu-DOTA-PP-γ-F11N: RP-HPLC (10→90% MeCN in H_2O with 0.1% TFA, 15 min, $\lambda = 220$ nm): $t_R = 7.41$ min, $K' = 3.39$; MS (ESI, positive): m/z calculated for $C_{90}H_{120}LuN_{19}O_{35}$: 2203.0, found: m/z = 1101.9 $[M+2H]^{2+}$.

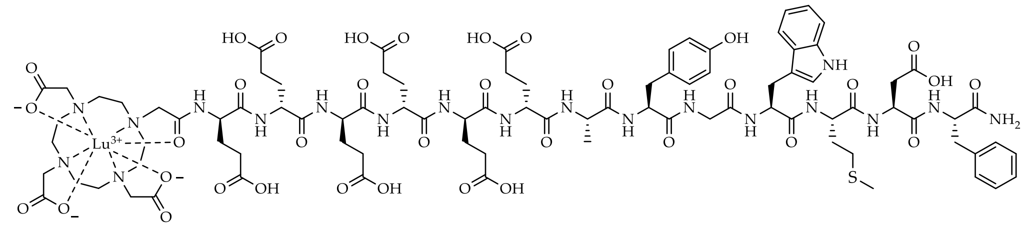

[natLu]Lu-CP04: RP-HPLC (10→90% MeCN in H_2O with 0.1% TFA, 15 min, $\lambda = 220$ nm): $t_R = 7.60$ min, $K' = 3.51$; MS (ESI, positive): m/z calculated for $C_{89}H_{118}LuN_{19}O_{35}S$: 2219.7, found: m/z = 1110.3 $[M+2H]^{2+}$.

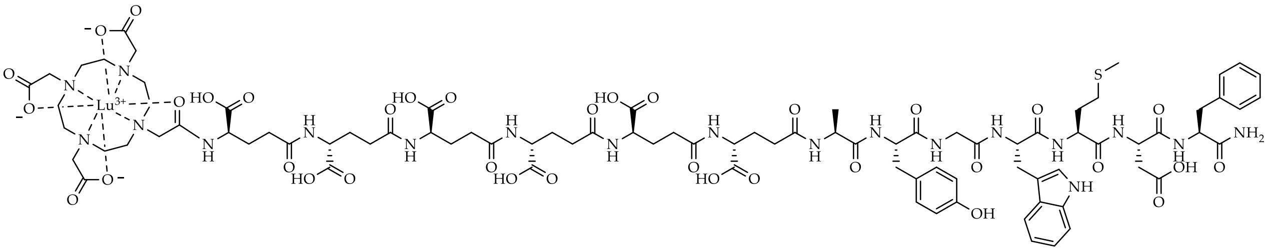

[natLu]Lu-γ-CP04: RP-HPLC (10→90% MeCN in H_2O with 0.1% TFA, 15 min, $\lambda = 220$ nm): $t_R = 7.40$ min, $K' = 3.39$; MS (ESI, positive): m/z calculated for $C_{89}H_{118}LuN_{19}O_{35}S$: 2219.7, found: m/z = 1110.8 $[M+2H]^{2+}$.

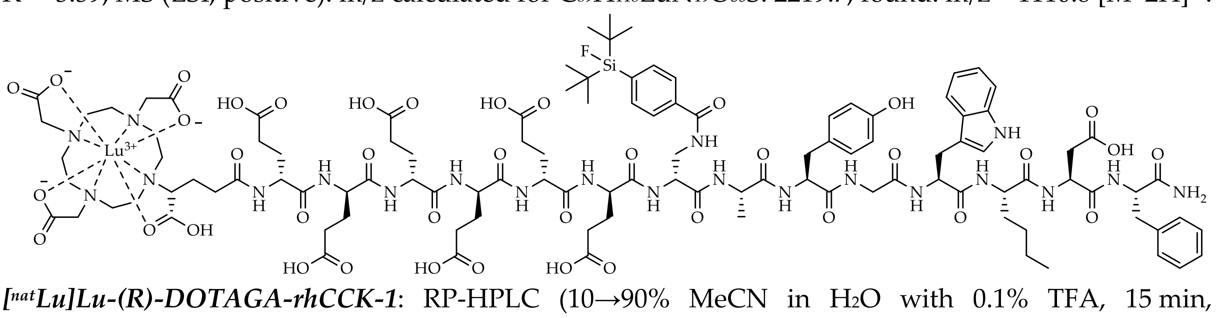

[natLu]Lu-(R)-DOTAGA-rhCCK-1: RP-HPLC (10→90% MeCN in H_2O with 0.1% TFA, 15 min, $\lambda = 220$ nm): $t_R = 10.1$ min, $K' = 4.99$; MS (ESI, positive): m/z calculated for $C_{111}H_{151}FLuN_{21}O_{39}Si$: 2625.6, found: m/z = 1312.8 $[M+2H]^{2+}$.

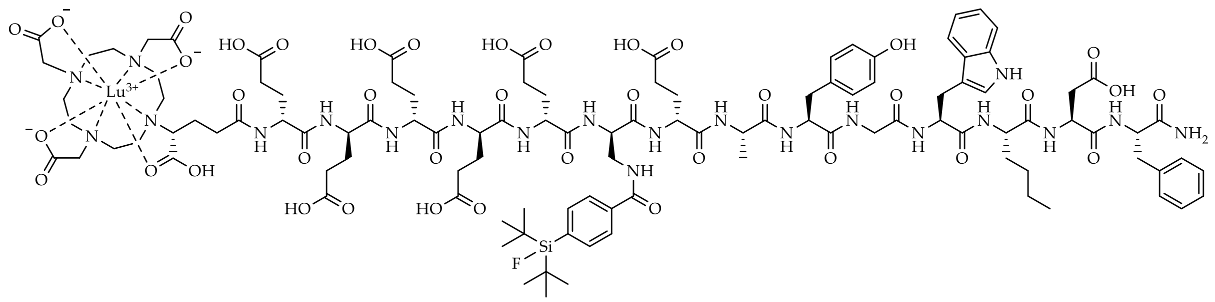

[natLu]Lu-(R)-DOTAGA-rhCCK-2: RP-HPLC (10→90% MeCN in H_2O with 0.1% TFA, 15 min, λ = 220 nm): t_R = 9.84 min, K′ = 4.83; MS (ESI, positive): m/z calculated for $C_{111}H_{151}FLuN_{21}O_{39}Si$: 2625.6, found: m/z = 1312.7 $[M+2H]^{2+}$.

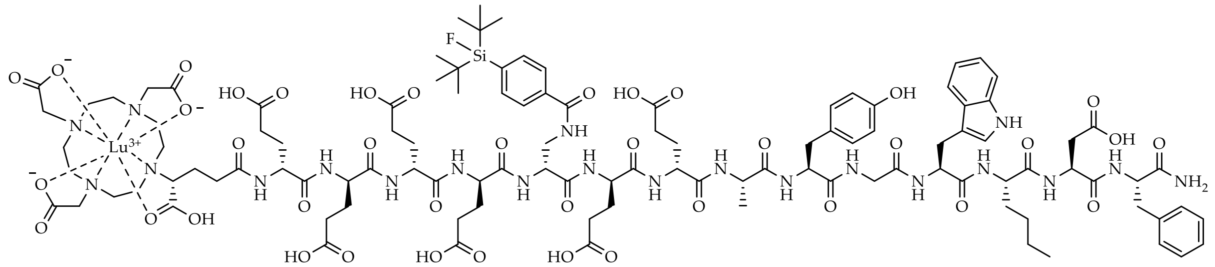

[natLu]Lu-(R)-DOTAGA-rhCCK-3: RP-HPLC (10→90% MeCN in H_2O with 0.1% TFA, 15 min, λ = 220 nm): t_R = 9.85 min, K′ = 4.84; MS (ESI, positive): m/z calculated for $C_{111}H_{151}FLuN_{21}O_{39}Si$: 2625.6, found: m/z = 1312.3 $[M+2H]^{2+}$.

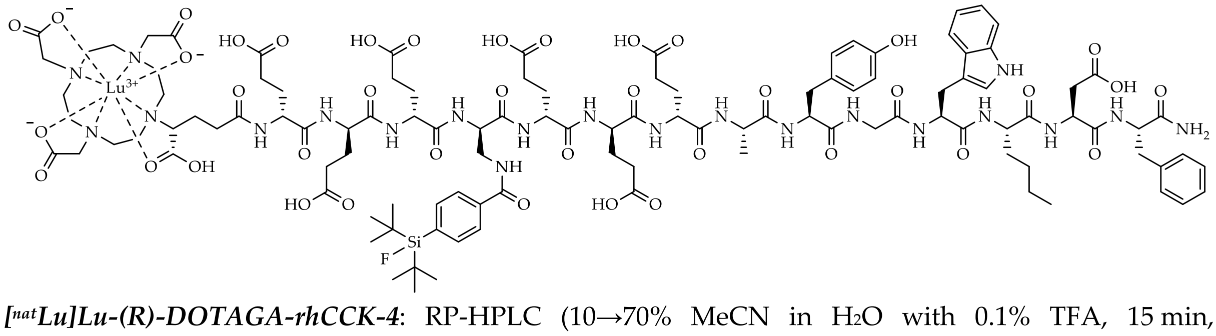

[natLu]Lu-(R)-DOTAGA-rhCCK-4: RP-HPLC (10→70% MeCN in H_2O with 0.1% TFA, 15 min, λ = 220 nm): t_R = 11.9 min, K′ = 6.05; MS (ESI, positive): m/z calculated for $C_{111}H_{151}FLuN_{21}O_{39}Si$: 2625.6, found: m/z = 1313.0 $[M+2H]^{2+}$.

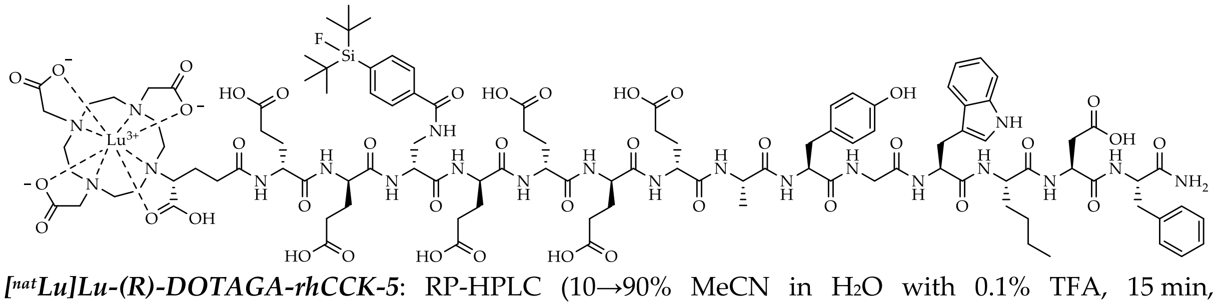

[natLu]Lu-(R)-DOTAGA-rhCCK-5: RP-HPLC (10→90% MeCN in H_2O with 0.1% TFA, 15 min, λ = 220 nm): t_R = 9.67 min, K′ = 4.73; MS (ESI, positive): m/z calculated for $C_{111}H_{151}FLuN_{21}O_{39}Si$: 2625.6, found: m/z = 1313.3 $[M+2H]^{2+}$.

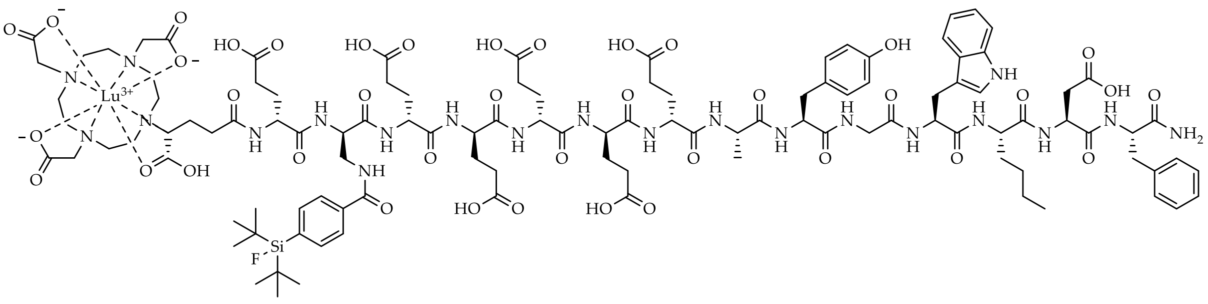

[natLu]Lu-(R)-DOTAGA-rhCCK-6: RP-HPLC (10→90% MeCN in H_2O with 0.1% TFA, 15 min, λ = 220 nm): t_R = 9.71 min, K′ = 4.76; MS (ESI, positive): m/z calculated for $C_{111}H_{151}FLuN_{21}O_{39}Si$: 2625.6, found: m/z = 1312.9 $[M+2H]^{2+}$.

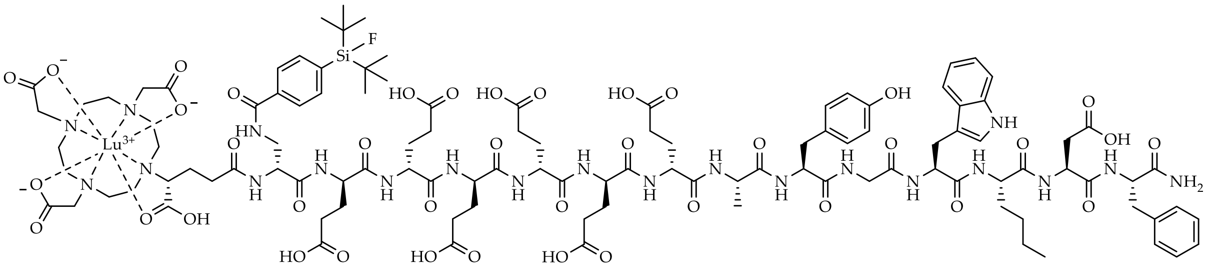

[natLu]Lu-(R)-DOTAGA-rhCCK-7: RP-HPLC (10→90% MeCN in H_2O with 0.1% TFA, 15 min, λ = 220 nm): t_R = 9.62 min, K′ = 4.70; MS (ESI, positive): m/z calculated for $C_{111}H_{151}FLuN_{21}O_{39}Si$: 2625.6, found: m/z = 1313.0 $[M+2H]^{2+}$.

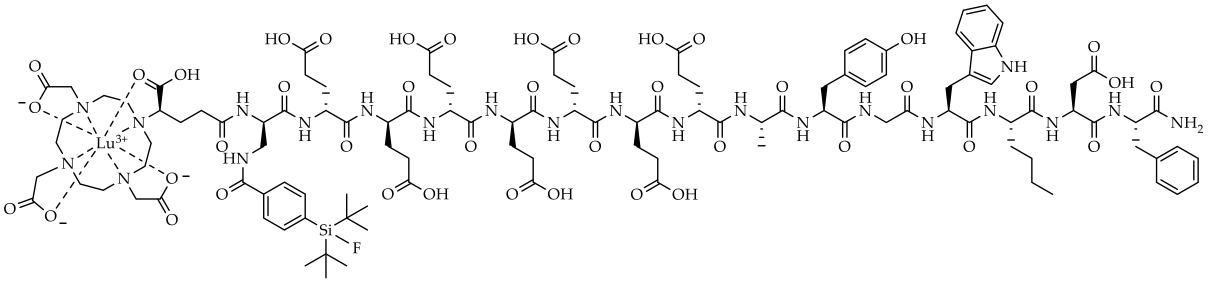

[natLu]Lu-(R)-DOTAGA-rhCCK-8: RP-HPLC (10→90% MeCN in H_2O with 0.1% TFA, 15 min, λ = 220 nm): t_R = 9.77 min, K′ = 4.79; MS (ESI, positive): m/z calculated for $C_{116}H_{158}FLuN_{22}O_{42}Si$: 2754.7, found: m/z = 1378.2 $[M+2H]^{2+}$.

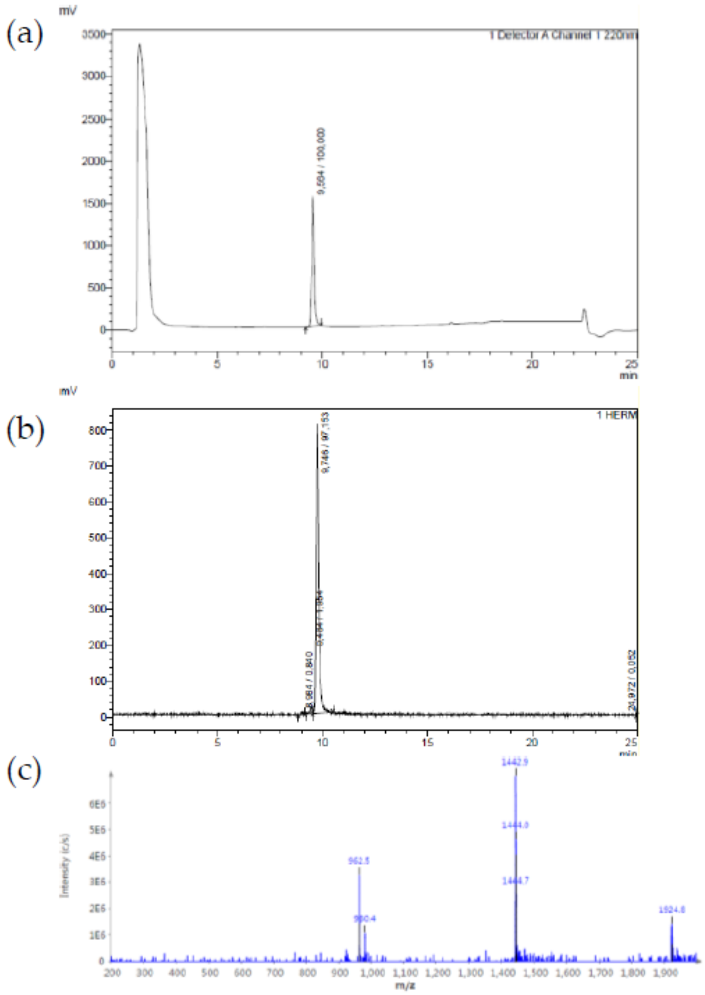

Figure S2. Confirmation of peptide identity and integrity for (**a**) [natLu]Lu-(*R*)-DOTAGA-rhCCK-9 and (**b**) [177Lu]Lu-(*R*)-DOTAGA-rhCCK-9 as analysed by analytical (radio-)RP-HPLC (MultoKrom 100-5 C18, 5 µm, 125 × 4.6 mm, CS Chromatographie GmbH, Langerwehe, Germany; 10→90% MeCN in H_2O + 0.1% TFA in 15 min). (**c**) Mass spectrum of [natLu]Lu-(*R*)-DOTAGA-rhCCK-9.

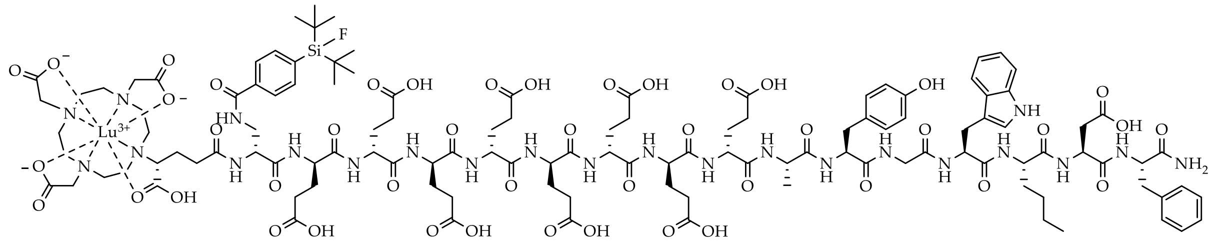

[natLu]Lu-(R)-DOTAGA-rhCCK-9: RP-HPLC (10→90% MeCN in H_2O with 0.1% TFA, 15 min, λ = 220 nm): t_R = 9.56 min, K′ = 4.67; MS (ESI, positive): m/z calculated for $C_{121}H_{165}FLuN_{23}O_{45}Si$: 2883.8, found: m/z = 1441.5 $[M+2H]^{2+}$.

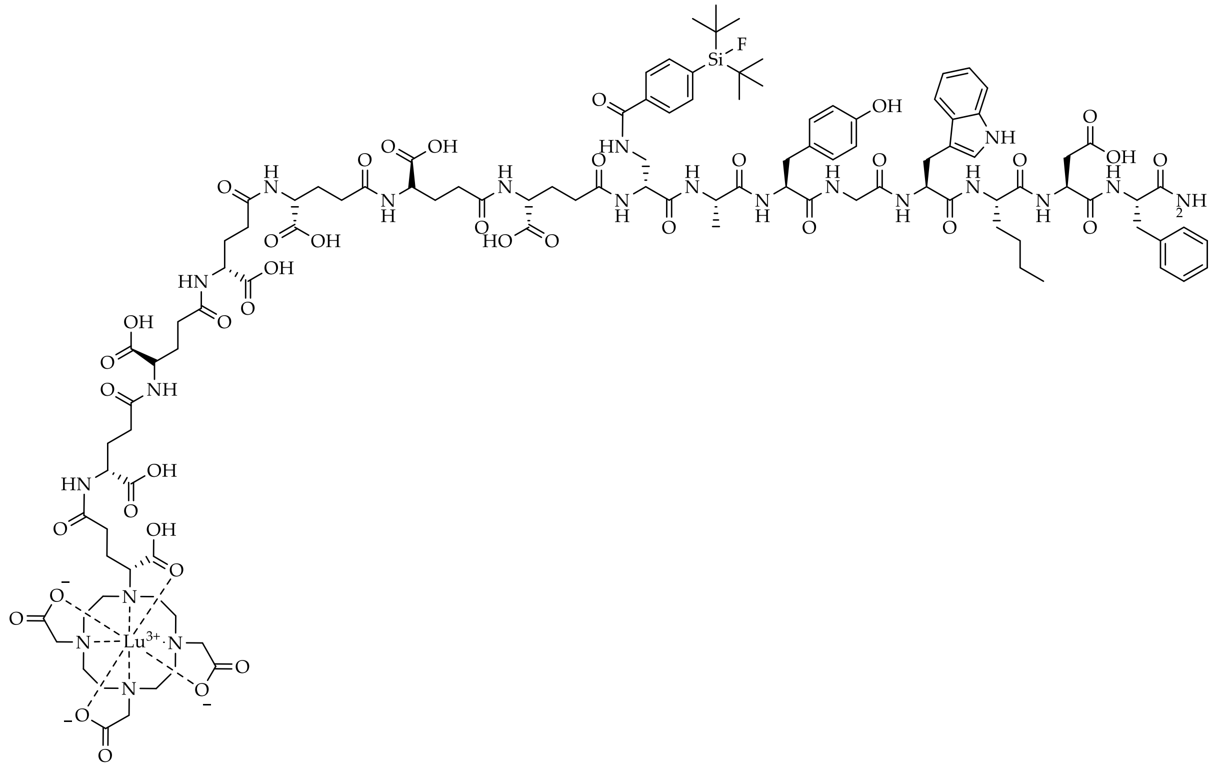

[natLu]Lu-(R)-DOTAGA-rhCCK-10: RP-HPLC (10→90% MeCN in H_2O with 0.1% TFA, 15 min, λ = 220 nm): t_R = 10.2 min, K′ = 5.05; MS (ESI, positive): m/z calculated for $C_{111}H_{151}FLuN_{21}O_{39}Si$: 2625.6, found: m/z = 1313.6 $[M+2H]^{2+}$.

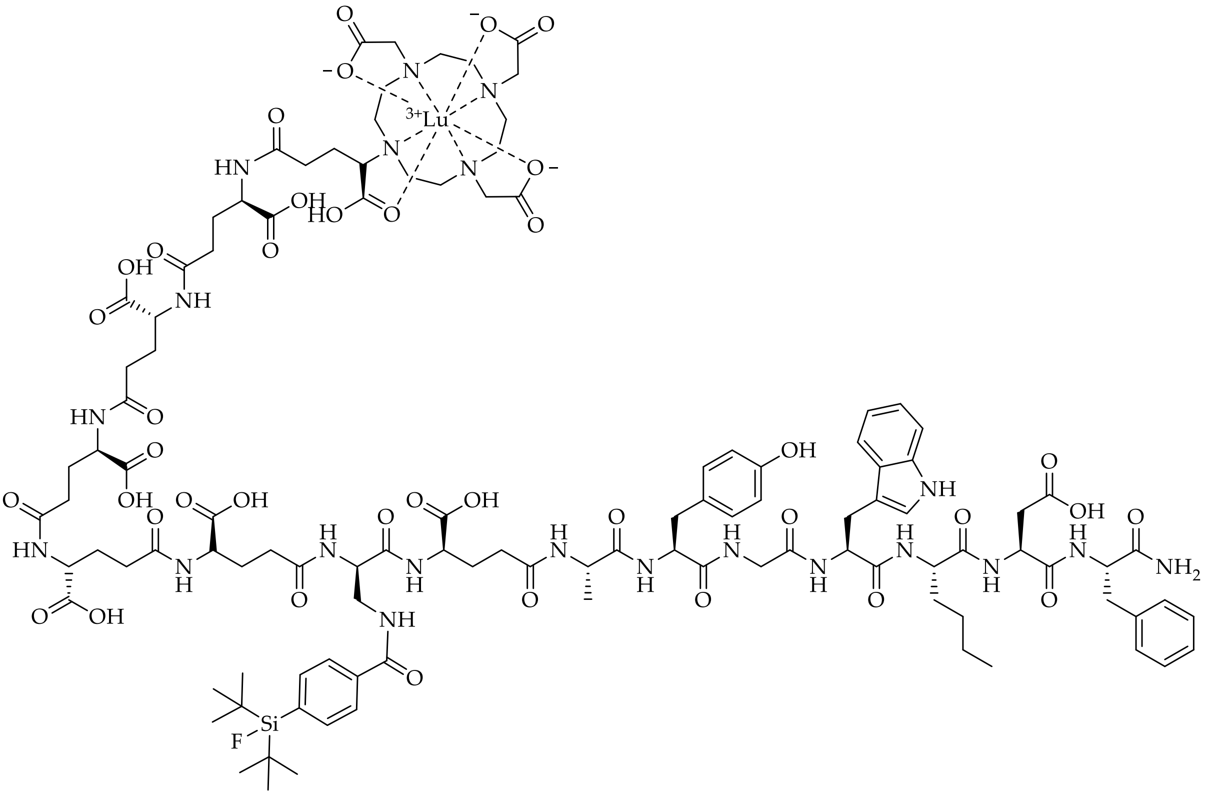

[natLu]Lu-(R)-DOTAGA-rhCCK-11: RP-HPLC (10→90% MeCN in H_2O with 0.1% TFA, 15 min, λ = 220 nm): t_R = 9.72 min, K′ = 4.76; MS (ESI, positive): m/z calculated for $C_{111}H_{151}FLuN_{21}O_{39}Si$: 2625.6, found: m/z = 1313.0 $[M+2H]^{2+}$.

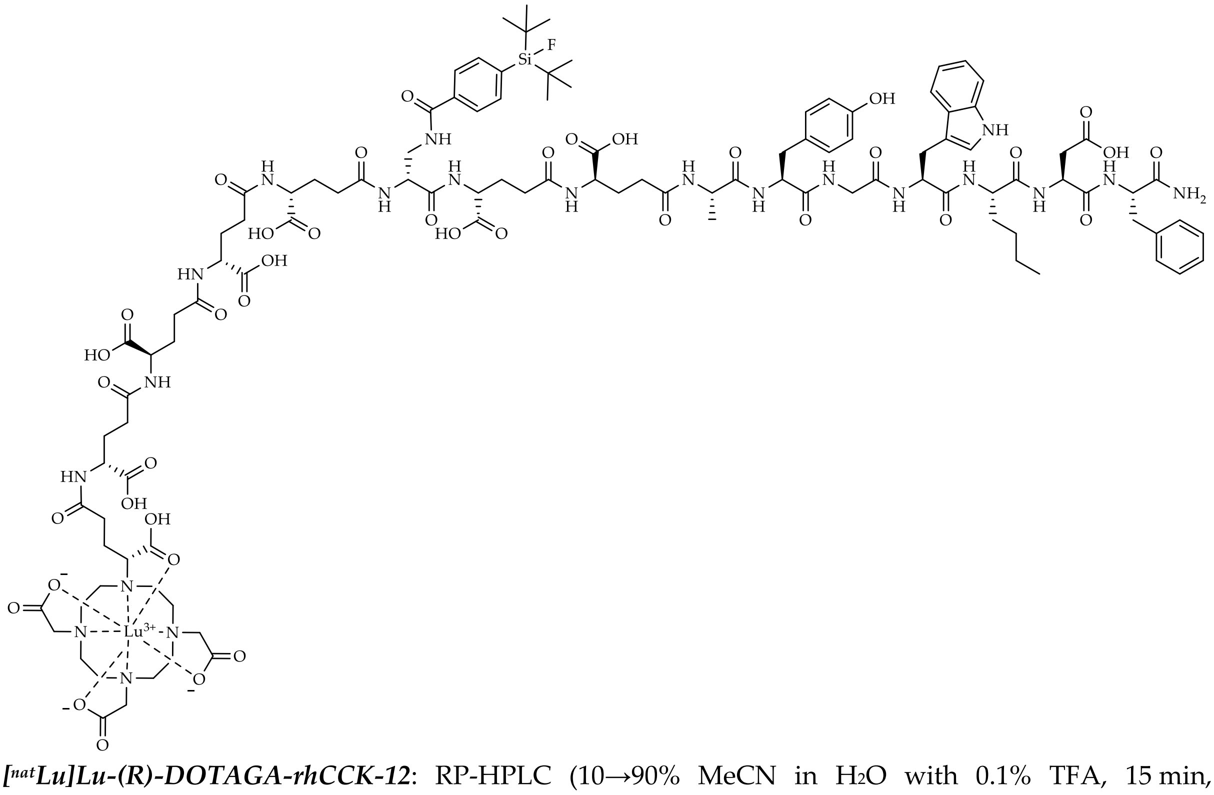

[natLu]Lu-(R)-DOTAGA-rhCCK-12: RP-HPLC (10→90% MeCN in H_2O with 0.1% TFA, 15 min, λ = 220 nm): t_R = 9.87 min, K′ = 4.85; MS (ESI, positive): m/z calculated for $C_{111}H_{151}FLuN_{21}O_{39}Si$: 2625.6, found: m/z = 1312.9 $[M+2H]^{2+}$.

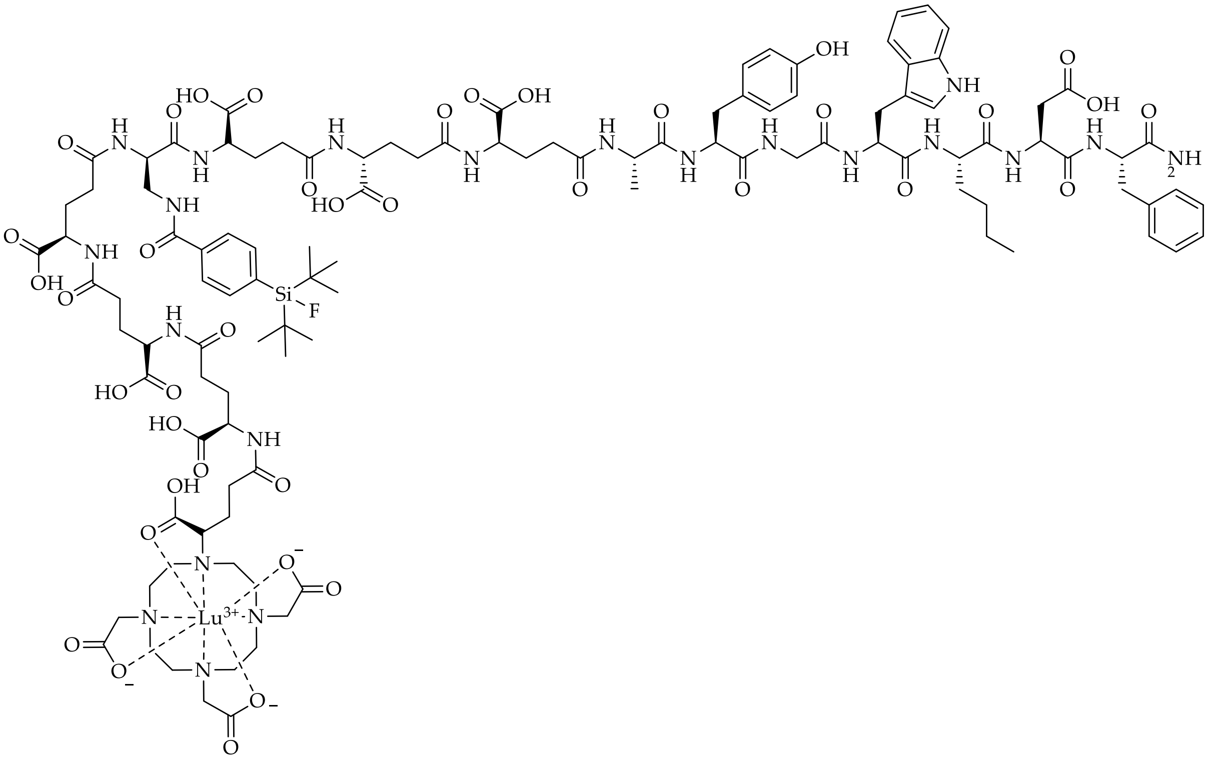

[natLu]Lu-(R)-DOTAGA-rhCCK-13: RP-HPLC (10→90% MeCN in H_2O with 0.1% TFA, 15 min, λ = 220 nm): t_R = 9.61 min, K′ = 4.70; MS (ESI, positive): m/z calculated for $C_{111}H_{151}FLuN_{21}O_{39}Si$: 2625.6, found: m/z = 1313.2 $[M+2H]^{2+}$.

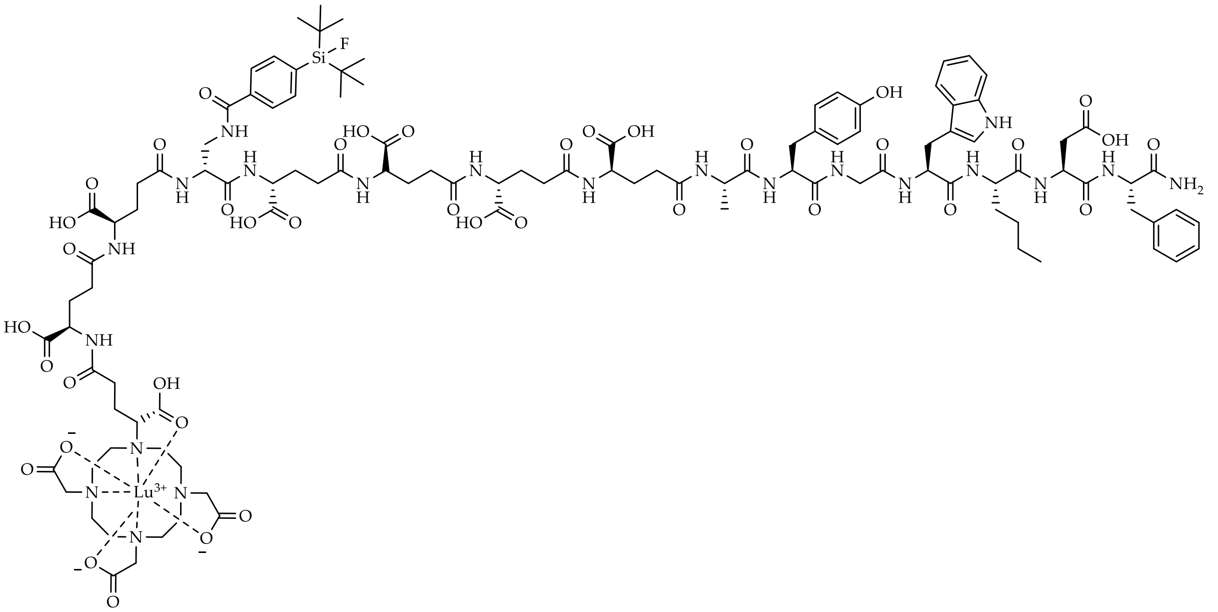

[natLu]Lu-(R)-DOTAGA-rhCCK-14: RP-HPLC (10→90% MeCN in H_2O with 0.1% TFA, 15 min, λ = 220 nm): t_R = 9.60 min, K′ = 4.69; MS (ESI, positive): m/z calculated for $C_{111}H_{151}FLuN_{21}O_{39}Si$: 2625.6, found: m/z = 1313.9 $[M+2H]^{2+}$.

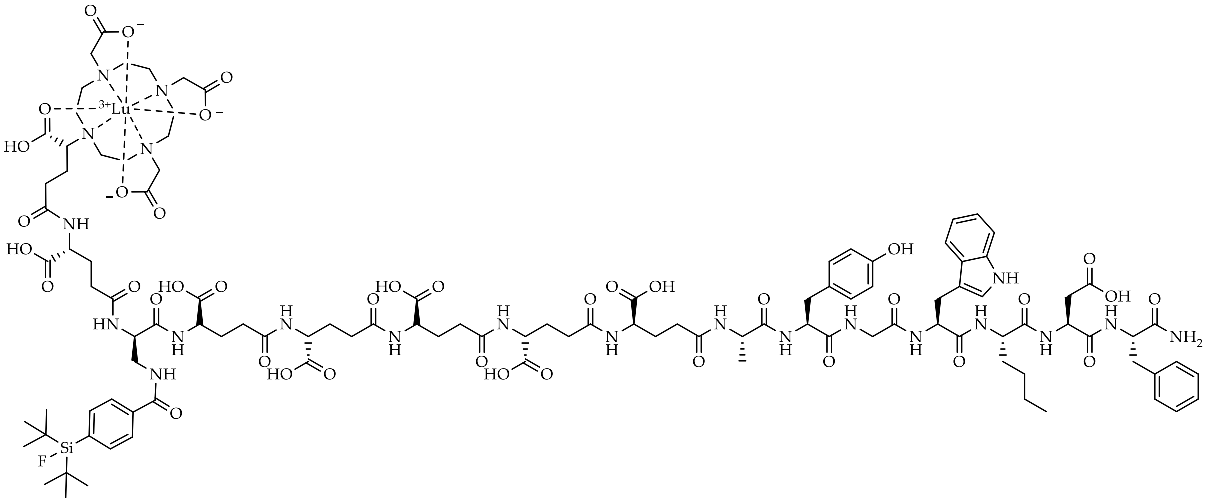

[^{nat}Lu]Lu-(R)-DOTAGA-rhCCK-15: RP-HPLC (10→90% MeCN in H_2O with 0.1% TFA, 15 min, λ = 220 nm): t_R = 9.62 min, K′ = 4.70; MS (ESI, positive): m/z calculated for $C_{111}H_{151}FLuN_{21}O_{39}Si$: 2625.6, found: m/z = 1313.3 $[M+2H]^{2+}$.

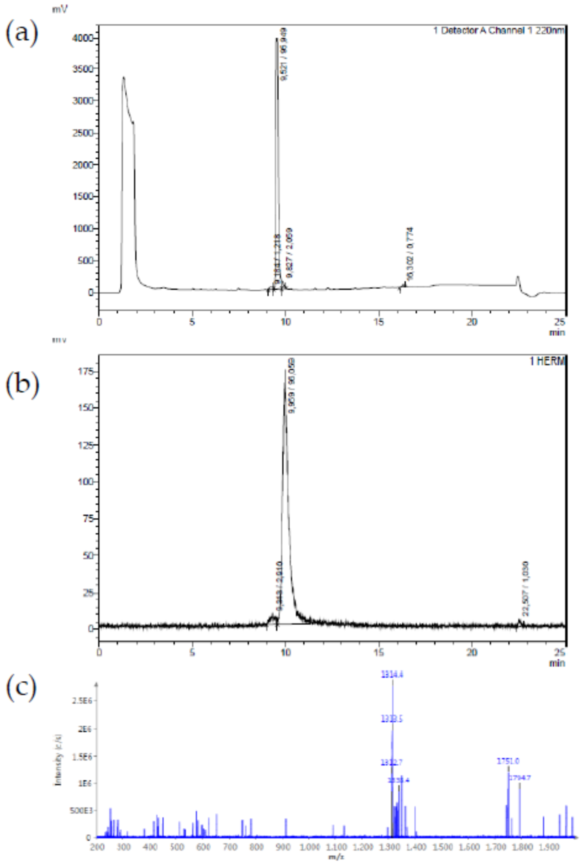

Figure S3. Confirmation of peptide identity and integrity for (**a**) [^{nat}Lu]Lu-(*R*)-DOTAGA-rhCCK-16 and (**b**) [^{177}Lu]Lu-(*R*)-DOTAGA-rhCCK-16 as analysed by analytical (radio-)RP-HPLC (MultoKrom 100-5 C18, 5 µm, 125 × 4.6 mm, CS Chromatographie GmbH, Langerwehe, Germany; 10→90% MeCN in H_2O + 0.1% TFA in 15 min). (**c**) Mass spectrum of [^{nat}Lu]Lu-(*R*)-DOTAGA-rhCCK-16.

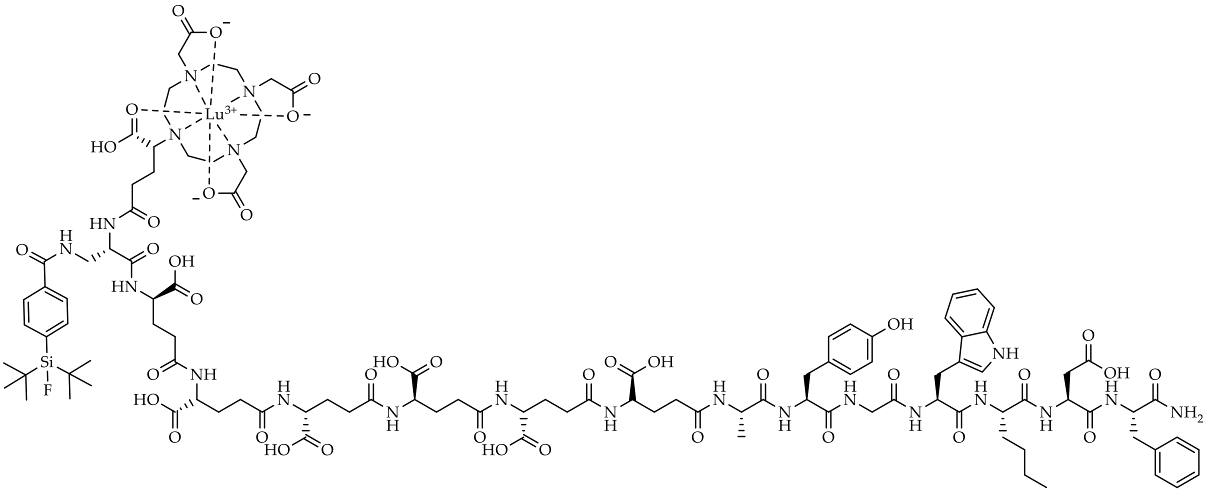

[natLu]Lu-(R)-DOTAGA-rhCCK-16: RP-HPLC (10→90% MeCN in H_2O with 0.1% TFA, 15 min, λ = 220 nm): t_R = 9.52 min, K′ = 4.64; MS (ESI, positive): m/z calculated for $C_{111}H_{151}FLuN_{21}O_{39}Si$: 2625.6, found: m/z = 1314.4 $[M+2H]^{2+}$.

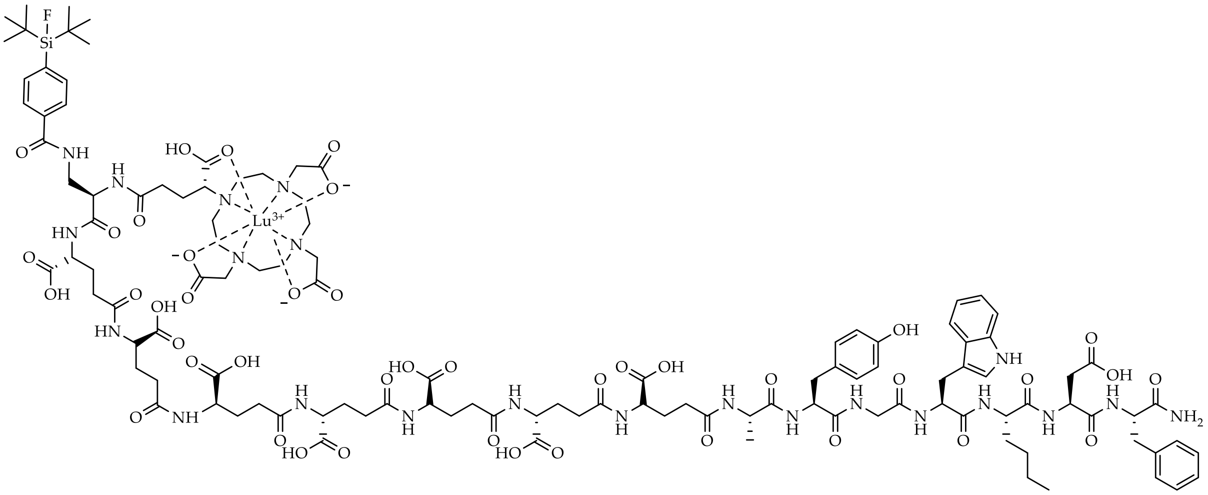

[natLu]Lu-(R)-DOTAGA-rhCCK-17: RP-HPLC (10→90% MeCN in H_2O with 0.1% TFA, 15 min, λ = 220 nm): t_R = 9.48 min, K′ = 4.62; MS (ESI, positive): m/z calculated for $C_{116}H_{158}FLuN_{22}O_{42}Si$: 2754.7, found: m/z = 1377.7 $[M+2H]^{2+}$.

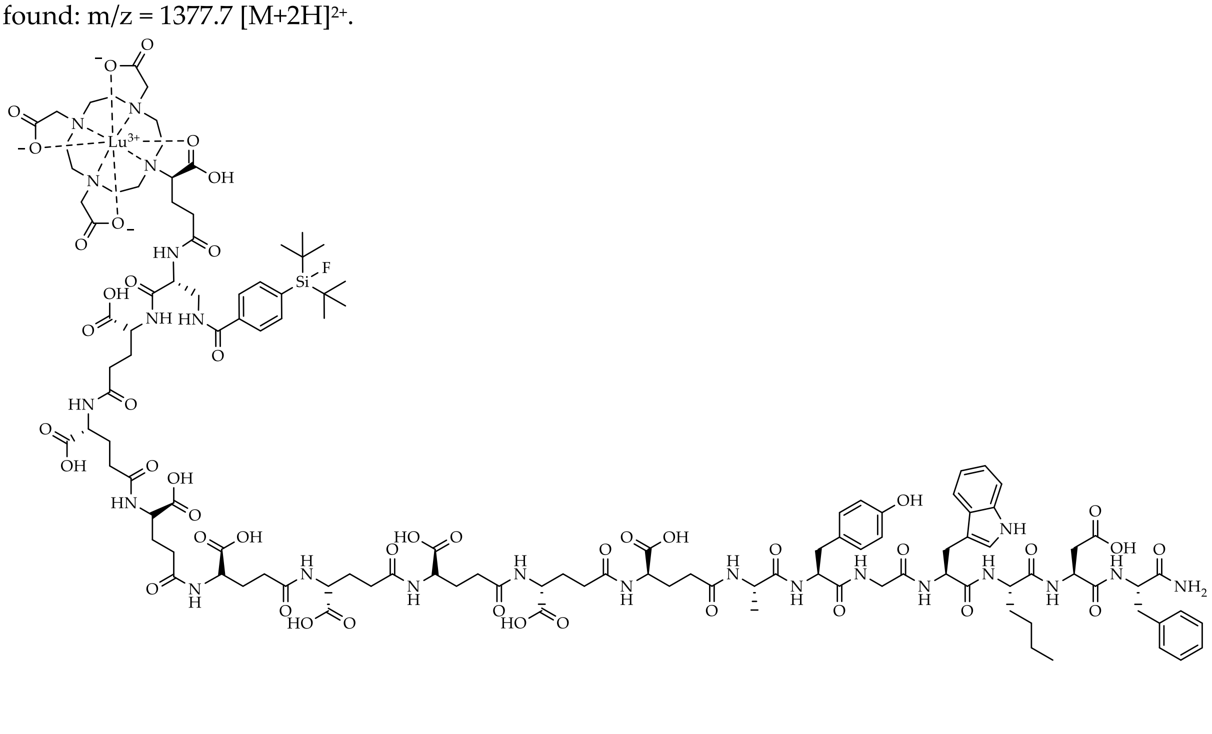

[^{nat}Lu]Lu-(R)-DOTAGA-rhCCK-18: RP-HPLC (10→90% MeCN in H_2O with 0.1% TFA, 15 min, λ = 220 nm): t_R = 9.50 min, K' = 4.63; MS (ESI, positive): m/z calculated for $C_{121}H_{165}FLuN_{23}O_{45}Si$: 2883.8, found: m/z = 1443.1 $[M+2H]^{2+}$.

^{177}Lu-labelling

For ^{177}Lu-labelling experiments, [^{177}Lu]$LuCl_3$ dissolved in hydrochloric acid (0.04 M, 40 GBq/mL) was acquired from *ITM Isotope Technologies Munich SE* (Garching, Germany). Radiolabelling of the peptide precursor (1 nmol) was performed at 90 °C for 15 min in a NaOAc-buffered (1 M, pH = 5.5) hydrochloric acid (0.04 M) solution. After radiolabelling, a radiolysis quencher (sodium ascorbate, 1 M in H_2O) was added and radiochemical purity was determined via radio-RP-HPLC and radio-TLC (instant thin layer chromatography paper impregnated with silica gel (iTLC-SG, *Agilent Technologies Inc.*, Folsom, United States); sodium citrate*1.5 H_2O (0.1 M)).

***In Vitro* Experiments**

Cell Culture. CCK-2R expressing rat pancreatic cancer cells AR42J (*CLS GmbH,* Eppelheim, Germany) were cultivated in monolayers in CELLSTAR® cell culture flasks acquired from *Greiner Bio-One GmbH* (Frickenhausen, Germany) at 37 °C in a humidified atmosphere (5% CO_2) using a HERAcell 150i-Incubator (*Thermo Fisher Scientific Inc.,* Waltham, United States). As nutrient medium RPMI 1640 medium, supplemented with 5 mM L-Gln 5 mL non-essential amino acids (100×) and 10% FCS, was used. Furthermore, a Dulbecco's PBS solution with 0.1% EDTA (*v/v*) was applied to detach the cells for cell passaging. The detached cells were counted using a Neubauer hemocytometer (*Paul Marienfeld*, Lauda-Königshofen, Germany). In addition, all operations under sterile conditions were accomplished using a MSC-Advantage safety workbench (*Thermo Fisher Scientific Inc.*, Waltham, United States).

Determination of IC_{50}. AR42J cells (2.0 × 10^5 cells/well) were seeded into 24-well plates 24 ± 2 h prior to testing, using 1 mL of nutrient medium (RPMI 1640, 5 mM L-Gln, 5 mL non-essential amino acids (100×), 10% FCS). Cells were incubated at 37 °C in a humidified atmosphere (5% CO_2).
After removal of the medium, each well was washed with 500 µL PBS. For the cell-based assay, 200 µL of nutrient medium (+5% BSA), [^{177}Lu]Lu-DOTA-PP-F11N (25 µL, 0.3 pmol) as a radiolabeled reference and 25 µL of the peptide of interest in increasing concentrations (10^{-10} to 10^{-4} M) in triplicate were added to the cells. Thereafter, the assay was incubated for 3 h at 37 °C and thereafter, the supernatant was collected. The cells were washed with 300 µL PBS and the collected supernatant fractions were unified. After lysis of the cells with NaOH (300 µL, 1 N) for 15 min, the respective wells were washed with NaOH (300 µL, 1 N) and both fractions were unified. The radioactivity of both, the supernatant and the lysed fractions was quantified using a γ-counter (*PerkinElmer Inc.,* Waltham, United States). The obtained data were evaluated *via* the GraphPad PRISM software (*GraphPad Software Inc*., La Jolla, United States), which calculates the halfmaximal inhibitory concentration (IC_{50}) of the peptides.

Internalisation Studies. For the determination of the internalisation kinetics of the various peptides, AR42J cells (3.0 × 10^5 cells/well) were seeded into polylysine coated 24-well plates adding 1 mL of nutrient medium. Afterwards, the cells were incubated for 24 ± 2 h at 37 °C in a humidified atmosphere (5% CO_2).
On the day of the experiment, the medium was removed, and each well was washed with nutrient medium (300 µL). Afterwards 200 µL of nutrient medium, 25 µL of the ^{177}Lu-labeled peptide (0.3 pmol, $n = 6$) and either 25 µL of nutrient medium for internalisation studies ($n = 3$) or 25 µL of [^{nat}Lu]Lu-DOTA-PP-F11N (10 µmol) for competition studies ($n = 3$) were added. Thereafter, the assay was incubated for various time points (1, 2, 4and 6 h) at 37 °C in a humidified atmosphere (5% CO_2). After incubation, the cells were put on ice for at least 1 min to stop internalisation kinetics and the supernatant was collected. Then, the cells were washed with an ice-cold nutrient medium (300 µL) and both fractions were unified. For the acid wash, 300 µL of an ice-cold glycine buffer (1 M, p1 M, pAfter lysis of the cells with NaOH (300 µL, 1 N) for 15 min, the respective wells were washed with NaOH (300 µL, 1 N) and both fractions were unified. The radioactivity of the supernatant, the acid wash and the lysed fractions were quantified using a γ-counter.

Table S1. Affinity and lipophilicity data of the compounds evaluated. Affinity data were determined on AR42J cells (2.0 × 10^5 cells/well) and [177Lu]Lu-DOTA-PP-F11N (0.3 pmol/well) as radiolabeled reference (3 h, 37 °C, RPMI 1640, 5 mM L-Gln, 5 mL non-essential amino acids (100×), 10% FCS + 5% BSA (*v*/*v*)).

**peptide**	***IC*$_{50}$** [nM]	**log*D*$_{7.4}$**
[nat/177Lu]Lu-DOTA-PP-F11N	12.8 ± 2.8	−4.75 ± 0.07
[nat/177Lu]Lu-DOTA-PP-γ-F11N	7.55 ± 0.48	−4.38 ± 0.05
[nat/177Lu]Lu-CP04	11.2 ± 0.2	−3.80 ± 0.10
[nat/177Lu]Lu-γ-CP04	8.88 ± 0.67	−4.30 ± 0.08
[nat/177Lu]Lu-(*R*)-DOTAGA-rhCCK-1	629 ± 48	−1.71 ± 0.10
[nat/177Lu]Lu-(*R*)-DOTAGA-rhCCK-2	156 ± 18	−1.95 ± 0.09
[nat/177Lu]Lu-(*R*)-DOTAGA-rhCCK-3	156 ± 4	−2.03 ± 0.10
[nat/177Lu]Lu-(*R*)-DOTAGA-rhCCK-4	185 ± 14	−2.19 ± 0.08
[nat/177Lu]Lu-(*R*)-DOTAGA-rhCCK-5	90.0 ± 2.3	−2.67 ± 0.05
[nat/177Lu]Lu-(*R*)-DOTAGA-rhCCK-6	56.8 ± 6.4	−2.22 ± 0.08
[nat/177Lu]Lu-(*R*)-DOTAGA-rhCCK-7	75.7 ± 10.7	−2.63 ± 0.08
[nat/177Lu]Lu-(*R*)-DOTAGA-rhCCK-8	58.0 ± 11.0	−2.19 ± 0.08
[nat/177Lu]Lu-(*R*)-DOTAGA-rhCCK-9	55.3 ± 7.8	−2.84 ± 0.04
[nat/177Lu]Lu-(*R*)-DOTAGA-rhCCK-10	863 ± 148	−2.15 ± 0.08
[nat/177Lu]Lu-(*R*)-DOTAGA-rhCCK-11	111 ± 13	−2.23 ± 0.07
[nat/177Lu]Lu-(*R*)-DOTAGA-rhCCK-12	89.7 ± 14.5	−2.34 ± 0.05
[nat/177Lu]Lu-(*R*)-DOTAGA-rhCCK-13	68.5 ± 11.4	−2.57 ± 0.06
[nat/177Lu]Lu-(*R*)-DOTAGA-rhCCK-14	51.3 ± 9.3	−2.26 ± 0.05
[nat/177Lu]Lu-(*R*)-DOTAGA-rhCCK-15	33.0 ± 11.1	−2.50 ± 0.02
[nat/177Lu]Lu-(*R*)-DOTAGA-rhCCK-16	20.4 ± 2.7	−2.54 ± 0.05
[nat/177Lu]Lu-(*R*)-DOTAGA-rhCCK-17	29.5 ± 1.9	−2.10 ± 0.06
[nat/177Lu]Lu-(*R*)-DOTAGA-rhCCK-18	20.4 ± 2.0	−2.16 ± 0.09

Table S2. Receptor-mediated internalisation values (37 °C, RPMI 1640, 5 mM L-Gln, 5 mL non-essential amino acids (100×), 10% FCS, 0.25 pmol/well) determined as percent (%) of the applied activity of [177Lu]Lu-(*R*)-DOTAGA-rhCCK-9 and -16 as well as the references [177Lu]Lu-DOTA-PP-F11N and [177Lu]Lu-CP04 using AR42J cells (3.0 × 10^5 cells/well) at different time points (1, 2, 4 and 6 h). Data are corrected for non-specific binding (10 µmol/well, [natLu]Lu-DOTA-PP-F11N).

**Peptide**	**Internalisation Values** (%)				**Internalisation Values*** (% of reference)
	**1 h**	**2 h**	**4 h**	**6 h**	**6 h**
[177Lu]Lu-DOTA-PP-F11N	6.44 ± 0.32	10.1 ± 0.4	17.5 ± 1.0	22.4 ± 0.6	-
[177Lu]Lu-CP04	5.16 ± 0.44	9.33 ± 0.36	15.4 ± 0.7	20.5 ± 0.5	91.6 ± 2.4
[177Lu]Lu-(*R*)-DOTAGA-rhCCK-9	2.08 ± 0.12	3.44 ± 0.55	8.01 ± 0.53	13.2 ± 0.5	65.7 ± 2.5
[177Lu]Lu-(*R*)-DOTAGA-rhCCK-16	7.63 ± 0.13	14.0 ± 1.3	23.2 ± 2.7	32.2 ± 2.1	135 ± 9

* Internalisation values are depicted as % of the reference [177Lu]Lu-DOTA-PP-F11N

Table S3. Total cell uptake (37 °C, RPMI 1640, 5 mM L-Gln, 5 mL non-essential amino acids (100×), 10% FCS, 0.25 pmol/well) determined as percent (%) of the applied activity of [^{177}Lu]Lu-(*R*)-DOTAGA-rhCCK-9 and -16 as well as the references [^{177}Lu]Lu-DOTA-PP-F11N and [^{177}Lu]Lu-CP04 using AR42J cells (3.0 × 10^5 cells/well) at different time points (1, 2, 4 and 6 h). Data are corrected for non-specific binding (10 µmol/well, [^{nat}Lu]Lu-DOTA-PP-F11N).

**Peptide**	**Total cell uptake** (%)			
	**1 h**	**2 h**	**4 h**	**6 h**
[^{177}Lu]Lu-DOTA-PP-F11N	7.03 ± 1.45	10.6 ± 1.5	18.5 ± 2.5	23.5 ± 1.4
[^{177}Lu]Lu-CP04	6.00 ± 0.42	10.0 ± 0.4	16.5 ± 0.9	20.4 ± 1.7
[^{177}Lu]Lu-(*R*)-DOTAGA-rhCCK-9	2.25 ± 0.23	3.46 ± 0.56	8.31 ± 0.59	13.4 ± 0.7
[^{177}Lu]Lu-(*R*)-DOTAGA-rhCCK-16	8.37 ± 0.07	14.4 ± 1.3	23.6 ± 2.8	32.8 ± 2.3

Table S4. Biodistribution data of [^{177}Lu]Lu-DOTA-PP-F11N, [^{177}Lu]Lu-(*R*)-DOTAGA-rhCCK-9 and [^{177}Lu]Lu-(*R*)-DOTAGA-rhCCK-16 in selected organs at 24 h p.i. in AR42J tumour-bearing CB17-SCID mice (100 pmol each). Data are expressed as %ID/g, mean ± SD.

**Organ**	**[^{177}Lu]Lu-DOTA-PP-F11N** (*n* = 4)	**[^{177}Lu]Lu-(*R*)-DOTAGA-rhCCK-9** (*n* = 4)	**[^{177}Lu]Lu-(*R*)-DOTAGA-rhCCK-16** (*n* = 4)	**[^{177}Lu]Lu-(*R*)-DOTAGA-rhCCK-16 competition studies** (*n* = 2)
Blood	0.00 ± 0.00	0.02 ± 0.00	0.01 ± 0.00	0.05 ± 0.00
Heart	0.02 ± 0.01	0.09 ± 0.03	0.11 ± 0.01	0.13 ± 0.01
Lung	0.03 ± 0.02	0.10 ± 0.05	0.09 ± 0.02	2.59 ± 1.27
Liver	0.67 ± 0.64	1.07 ± 0.54	1.18 ± 0.27	2.31 ± 0.66
Spleen	0.07 ± 0.03	0.70 ± 0.69	0.62 ± 0.03	1.73 ± 0.01
Pancreas	0.05 ± 0.01	0.31 ± 0.20	0.39 ± 0.12	0.20 ± 0.01
Stomach	0.36 ± 0.05	1.80 ± 0.37	3.51 ± 0.50	0.15 ± 0.04
Intestine	0.04 ± 0.02	0.26 ± 0.04	0.20 ± 0.04	0.16 ± 0.03
Kidney	3.08 ± 0.51	84.4 ± 22.7	85.5 ± 11.3	105 ± 21
Adrenal	0.03 ± 0.03	0.45 ± 0.30	1.49 ± 1.50	0.40 ± 0.11
Muscle	0.00 ± 0.00	0.03 ± 0.01	0.05 ± 0.01	0.06 ± 0.04
Bone	0.03 ± 0.01	0.82 ± 0.42	0.17 ± 0.02	0.24 ± 0.04
Tumour	1.88 ± 0.82	6.40 ± 1.48	15.70 ± 3.27	0.91 ± 0.04

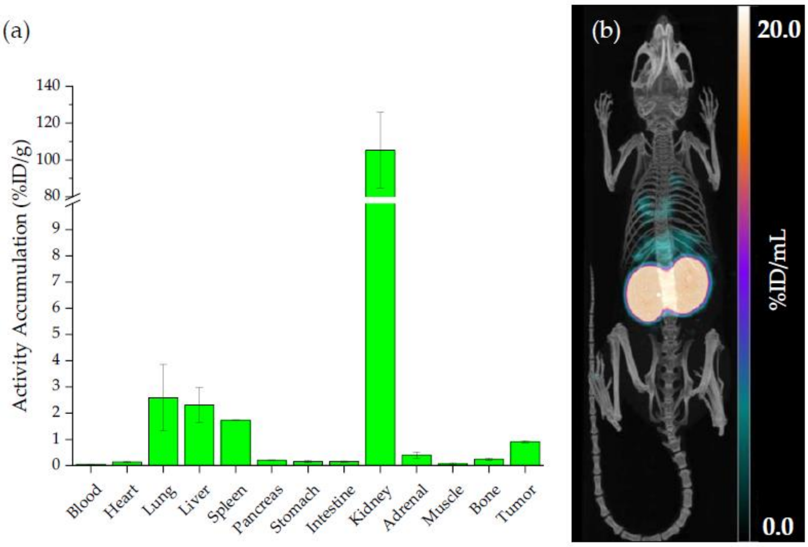

Figure S4. (**a**) Biodistribution of [^{177}Lu]Lu-DOTA-rhCCK-16 (100 pmol) co-injected with [^{nat}Lu]Lu-DOTA-MGS5 (40 nmol) in selected organs (%ID/g) at 24 h p.i. in AR42J tumour-bearing CB17-SCID mice. Data is expressed as mean ± SD (*n* = 2). (**b**) Representative *μ*SPECT/CT images of [^{177}Lu]Lu-DOTA-rhCCK-16 co-injected with [^{nat}Lu]Lu-DOTA-MGS5 (40 nmol) at 24 h p.i. in AR42J tumour-bearing CB17- SCID mice.
